# Ethnopharmacological Approaches for Dementia Therapy and Significance of Natural Products and Herbal Drugs

**DOI:** 10.3389/fnagi.2018.00003

**Published:** 2018-02-12

**Authors:** Devesh Tewari, Adrian M. Stankiewicz, Andrei Mocan, Archana N. Sah, Nikolay T. Tzvetkov, Lukasz Huminiecki, Jarosław O. Horbańczuk, Atanas G. Atanasov

**Affiliations:** ^1^Department of Pharmaceutical Sciences, Faculty of Technology, Kumaun University, Nainital, India; ^2^Institute of Genetics and Animal Breeding of the Polish Academy of Sciences, Jastrzebiec, Poland; ^3^Department of Pharmaceutical Botany, Iuliu Hațieganu University of Medicine and Pharmacy, Cluj-Napoca, Romania; ^4^ICHAT and Institute for Life Sciences, University of Agricultural Sciences and Veterinary Medicine, Cluj-Napoca, Romania; ^5^Department of Molecular Biology and Biochemical Pharmacology, Institute of Molecular Biology Roumen Tsanev, Bulgarian Academy of Sciences, Sofia, Bulgaria; ^6^Department of Pharmacognosy, University of Vienna, Vienna, Austria

**Keywords:** Alzheimer's disease, amyloid fibrils, β-amyloid, dementia, ethnopharmacology, herbal drugs

## Abstract

Dementia is a clinical syndrome wherein gradual decline of mental and cognitive capabilities of an afflicted person takes place. Dementia is associated with various risk factors and conditions such as insufficient cerebral blood supply, toxin exposure, mitochondrial dysfunction, oxidative damage, and often coexisting with some neurodegenerative disorders such as Alzheimer's disease (AD), Huntington's disease (HD), and Parkinson's disease (PD). Although there are well-established (semi-)synthetic drugs currently used for the management of AD and AD-associated dementia, most of them have several adverse effects. Thus, traditional medicine provides various plant-derived lead molecules that may be useful for further medical research. Herein we review the worldwide use of ethnomedicinal plants in dementia treatment. We have explored a number of recognized databases by using keywords and phrases such as “dementia”, “Alzheimer's,” “traditional medicine,” “ethnopharmacology,” “ethnobotany,” “herbs,” “medicinal plants” or other relevant terms, and summarized 90 medicinal plants that are traditionally used to treat dementia. Moreover, we highlight five medicinal plants or plant genera of prime importance and discuss the physiological effects, as well as the mechanism of action of their major bioactive compounds. Furthermore, the link between mitochondrial dysfunction and dementia is also discussed. We conclude that several drugs of plant origin may serve as promising therapeutics for the treatment of dementia, however, pivotal evidence for their therapeutic efficacy in advanced clinical studies is still lacking.

## Introduction

Dementia is a clinical syndrome wherein gradual decline of mental and cognitive capabilities of an afflicted person takes place. As the disease progresses, the ability to function independently of an affected individual deteriorates due to memory loss (Burgess et al., 2002; Damasio and Gabrowski, 2004; Grand et al., 2011). The causes of dementia can be either reversible or irreversible. The reversible causes include, for example, substance abuse, subdural hematoma, removable tumors, and central nervous system (CNS) infections (Tripathi and Vibha, 2009). Some of the irreversible causes of dementia are neurodegenerative diseases such as Alzheimer's disease (AD), Parkinson's disease (PD), and Huntington's disease (HD) (Mehan et al., 2012).

Thus, dementia is associated with multiple predisposing conditions and risk factors, among which aging is the greatest and most obvious one (Blennow et al., 2006; Corrada et al., 2010). The most widespread group of dementias is related to neurodegenerative disorders, including AD, PD, and HD, or amyotrophic lateral sclerosis (ALS). Another important group of dementias are vascular cognitive impairments. These pathologies often coexist with neurodegenerative dementias (Iadecola, 2013). Vascular cognitive impairments arise due to various cerebrovascular pathologies, such as hypoperfusions or hemorrhages causing disruption of the blood-brain barrier (BBB) and neurovascular units, usually in hemispheric white matter (Iadecola, 2013). Other diseases may also contribute to development of dementia. Such diseases include metabolic disorders, which participate in the pathology via dysregulation of energy management (Cai et al., 2012), AIDS, which causes indirect damage to the brain through immune activated macrophages (Navia and Rostasy, 2005), or even systemic infections (Lim et al., 2015). Various environmental factors also increase the risk of developing dementia. For example toxins contained in abused substances (Ridley et al., 2013), pesticides (Yan et al., 2016), or air pollution (Rivas-Arancibia et al., 2013; Power et al., 2016) may cause oxidative stress and subsequent neuronal cell death. In addition to the above factors, the etiology of dementia includes a genetic component. The occurrence of dementia is connected with numerous gene polymorphisms and other mutations (Weksler et al., 2013). For example, mutations in three deterministic autosomal dominant genes, i.e., presenilin 1 (PSEN1) on chromosome 14q, presenilin 2 (PSEN2) on 1q, and amyloid precursor protein (APP) on 21q, are associated with early-onset AD (EOAD) (Giri et al., 2016). The apolipoprotein E (APOE) gene, located at locus 19q13.2, is the strongest genetic risk factor for sporadic lead-onset AD (LOAD) (Corder et al., 1993; Giri et al., 2016; Swerdlow et al., 2017). There are three common APOE alleles, namely APOE ε2, ε3, and ε4 alleles, among which the APOE ε4 genotype is mainly associated with the higher risk of AD development (Mahley and Rall, 2000; Giri et al., 2016; Swerdlow et al., 2017). The link between the APOE ε4 genotype and development of AD pathology is complex (Giri et al., 2016). Studies suggest that APOE ε4 is associated with 3- up to 12-fold increased risk of LOAD and earlier onset of dementia in individuals with PSEN1 mutation, whereas APOE ε2 decreases the risk of LOAD (Pastor et al., 2003; Giri et al., 2016). In addition, APOE ε4 contributes to AD pathogenesis by Aβ-independent mechanisms involving neurovascular functions, synaptic plasticity, cholesterol homeostasis, and neuroinflammation (Giri et al., 2016). Thus, the presence of APOE4 ε4 allele is considered one of the risk factors for AD; more specifically, it is associated with increased risk of cerebral amyloid angiopathy and age-related cognitive decline during aging (Liu et al., 2013).

Dementia is a highly prevalent syndrome. Despite its prevalence, some evidence suggest that only 10% (low-middle-income countries) to 50% (high-income countries) of all dementia cases are diagnosed (Prince et al., 2016). In addition, the number of dementia cases will grow in consecutive years, as dementia mostly affects the elderly, and the number of people with advanced age is rising rapidly due to the global increase in life expectancy. Quaglio et al. (2016) approximate, that 1.5–2% of the Europeans are currently affected by dementia. On a similar note, Prince et al. (2016) estimated that in 2015 around 47 million people globally suffered from dementia. This number may reach roughly 131 million in 2050 (Prince et al., 2016). In the year 2021, one million people will be affected by dementia in the UK alone (Knapp et al., 2007). The importance of developing novel dementia treatments was recognized by the G7 summit in December 2014. The forum participants recommended that dementia should be treated as a global priority with the main objective to introduce effective therapy by 2025 (Cummings et al., 2016).

Dementia is a significant burden on society both by eliciting human suffering and financially. Dementia is the fifth most frequent cause of death in high-income countries (Dolgin, 2016). Their caregivers were also negatively affected by the health conditions of the patients and showed moderately high levels of depressive symptoms (Schulz et al., 2008). According to the World Alzheimer Report 2016 (Prince et al., 2016), not only in the low-income countries, but also in the high-income countries people with dementia have poor access to healthcare due to its high cost and ineffective diagnostic systems. Dementia management is very expensive due to long-lasting and costly care that the patients receive (Hurd et al., 2013). Various costs related to dementia, including health services, social services, unpaid careers, and others reach around €23 billion per year alone in the UK (Luengo-Fernandez et al., 2010). This economic burden can be further illustrated by the fact that, in the UK, the current annual cost of dementia is higher than current annual costs of heart disease and cancer combined (Luengo-Fernandez et al., 2010). Another prediction suggests that by 2050 dementia and Alzheimer's disease may cost the United States alone around USD 1 trillion (Dolgin, 2016).

In this review, we present a global overview on the worldwide use of ethnomedicine for the management of dementia. Moreover, we also focus on five prominent plants traditionally used for dementia treatment and highlight the constituents, which may be responsible for plant's bioactivity.

Currently, there is no highly effective medicine that stops the progressive course of dementia (Abbott, 2011). We propose that learning about the active substances produced by some plants and their mechanism of action may lead to the development of novel therapies for dementia. The natural products pool represents a continuous major source for drug discovery (Atanasov et al., 2015). In this context, the present review could serve as a useful resource for the development of ethnomedicine-derived pharmaceuticals for the dementia therapy.

## Frequent forms of dementia

The most common types of dementia are AD-related dementia (approximately 50–80% of all dementia cases) (Qiu et al., 2009; Abbott, 2011), vascular dementia (approximately 20–30%) (Abbott, 2011; Iadecola, 2013), dementia with Lewy bodies (between 15 and 35% according to Zupancic et al., 2011, or less than 5% according to Abbott, 2011) and the frontotemporal dementia (FTD), which is the fourth most frequent form of presenile dementia (between 5 and 10%) (Abbott, 2011; Table 1).

**Table 1:** Most common forms of dementia (according to Abbott, 2011).

**Dementia form**	**Neuropathology**	**Symptoms**	**Dementia cases (%)**
AD-related dementia	Aβ plaques, neurofibrillary tangles	Memory deficits, depression, poor judgment or evidence of mental confusion	50–80
Vascular dementia	Decreased or interrupted blood flow to the brain, hypoperfusion, oxidative stress	Similar to AD, but less affected memory	20–30
Dementia with Lewy bodies	α-Synuclein aggregates in neurons and glial cells (cortical Lewy bodies)	Similar to AD and less to PD, hallucinations, tremors, impaired attention	<5
Frontotemporal dementia	Accumulation of MAP tau, atrophy of frontal and temporal lobes	Changes in social behavior, difficulties with language	5–10

Common to these dysfunctions is a presence of abnormal accumulated proteins in the brains of patients. For example, in AD amyloid beta (Aβ) peptides aggregate into amyloid plaques (Hardy and Higgins, 1992), while TDP-43 protein accumulates in the human brain during the course of FTD (Baloh, 2011). Brains of FTD patients show gross atrophy of frontal and anterior temporal lobes (Brun, 1987; McKhann et al., 2001), and their histopathology reveals microvacuolar degeneration and loss of pyramidal neurons in the frontal and temporal cortex (Rabinovici and Miller, 2010). Furthermore, pathologic accumulation of microtubule-associated protein (MAP) tau is a process also common for AD and FTD (Iqbal et al., 2016). Abnormal hyperphosphorylation of tau protein leads to its aggregation into intraneuronal neurofibrillary tangles (Iqbal et al., 2010). Vascular dementia is a heterogeneous group of brain disorders associated with a cognitive impairment that is attributed to multifactorial cerebrovascular pathologies, such as hypoperfusion, oxidative stress, and inflammation (Iadecola, 2013). Dementia with Lewy bodies is characterized by the presence of α-synuclein aggregates in neurons and glial cells (Zupancic et al., 2011), and also associated with cholinergic as well as glutamate transmission deficiencies (Zupancic et al., 2011). It may also be concluded, that there is a great deal of overlap between the symptoms of different types of dementia (Abbott, 2011).

Naturally, the pathology of these diseases is more complicated (Blennow et al., 2006). Various markers of AD can be found in the brains of afflicted patients (Blennow et al., 2006). These markers include, e.g., dysregulation of signaling of memory-related neurotransmitter acetylcholine (ACh) (Kihara and Shimohama, 2004), vascular damage (Franzblau et al., 2013), loss of neurons (Niikura et al., 2006) and synapses (Shankar and Walsh, 2009). Mitochondrial dysfunction is another pathology, which was recognized as an important early event in the AD progression and, therefore, may be considered as promising target for the treatment of AD and AD-related dementia (Kumar and Singh, 2015). We briefly describe the mitochondrial dysfunction in the context of dementia in section Mitochondrial Dysfunction and Neurodegeneration.

## Mitochondrial dysfunction and neurodegeneration

**Figure 1:**
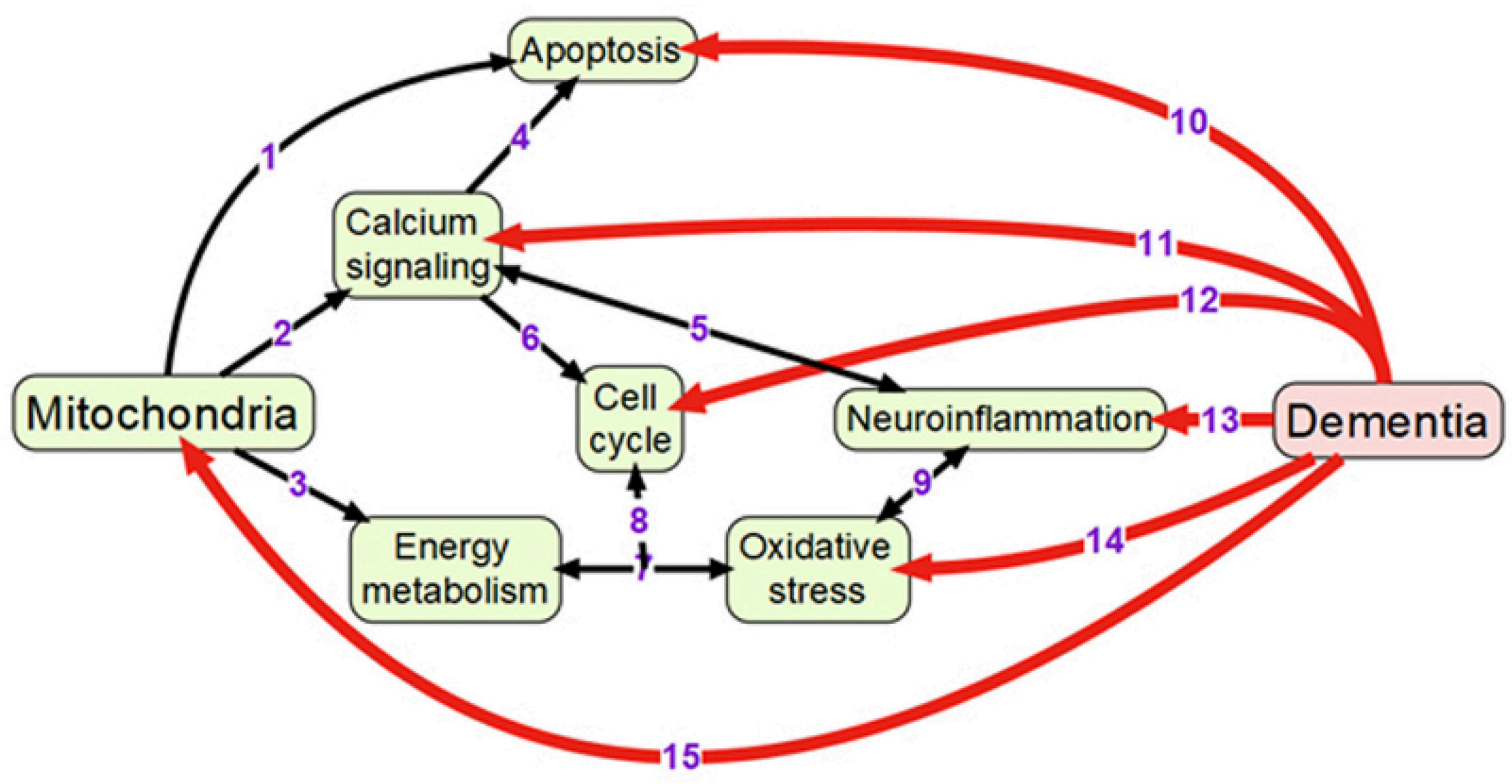
Overview of mechanisms linking mitochondrial activity with dementia: (1) Mitochondria are crucially important for activating apoptosis (Wang and Youle, 2009); (2) Mitochondria regulate calcium signaling pathway (Walsh et al., 2009); (3) Oxidative phosphorylation occurs in electron transport chain of mitochondria; (4) Calcium signaling induces apoptosis (Hajnoczky et al., 2003); (5) Calcium and neuroinflammatory signaling pathways interact with each other (Sama and Norris, 2013); (6) Cell cycle requires calcium signaling (Berridge, 1995); (7) Mitochondrial oxidative phosphorylation is one of the main sources of reactive oxygen species (Dai et al., 2014); (8) Oxidative phosphorylation and reactive oxygen species regulate cell cycle (Antico Arciuch et al., 2012); (9) Oxidative stress and neuroinflammation are highly interconnected processes (Gao et al., 2014); (10) Neuronal apoptosis (LeBlanc, 2005; Favaloro et al., 2012); (11) Impaired calcium signaling (Berridge, 2011; Nimmrich and Eckert, 2013); (12) Changes in cell cycle (Raina et al., 2000; Katsel et al., 2013); (13) Presence of neuroinflammation (Pasqualetti et al., 2015); (14) Increased oxidative stress (Bennett et al., 2009; Kumar and Singh, 2015); (15) Changes in mitochondrial morphology and functions (Spano et al., 2015; Hung et al., 2018).

Mitochondria are double membrane-enclosed organelles that are responsible to exert a broad range of cellular functions that include the most important adenosine triphosphate (ATP) production (energy conversion), but also involvement in several homeostatic processes, such as regulation of cell cycle and cell growth, calcium handling, and apoptosis-programmed cell death (van Horssen et al., 2017). Moreover, mitochondria play a crucial role in many other essential metabolic processes (van Horssen et al., 2017). Thus, mitochondrial diseases often have an associated metabolic component and, therefore, mitochondrial defects are predictable in energy-dependent disturbances, inflammation, and aging (Chan, 2006; Banasch et al., 2011). Hence, mitochondrial dysfunction is one of the key pathological features in various age-related neurodegenerative diseases including AD-associated dementia due to the pivotal role of mitochondria in neuronal cell survival or death (Moreira et al., 2010). For example, it has been proposed that mitochondrial network remodeling plays a prominent role in neurodegeneration (Zhu et al., 2013; Burte et al., 2015). The mitochondrial cascade hypothesis proposed the mitochondrial dysfunction as a principal episode in AD pathology (Moreira et al., 2010; Swerdlow et al., 2010). Moreover, mitochondrial dysfunction, which is also described as an impairment of electron transport chain, is responsible to increase the production of reactive oxagen species (ROS) and change mitochondrial dynamics (Beal, 2005; Lin and Beal, 2006; Hung et al., 2018). A recent study has shown the reciprocal relationship between ROS and mitochondrial dynamics during early stages of neurodegeneration (Hung et al., 2018). It has also been found that the cause for mitochondria-mediated toxicity is the progressive accumulation of Aβ in mitochondria (Chen and Yan, 2010). Recent studies provided the crucial role of mitochondrial dysfunction in regulating the ROS and intracellular calcium levels in neuronal cells (Aminzadeh et al., 2018). In addition, Lee et al. investigated the relationship between the NAD-dependent deacetylase sirtuin-3 (SIRT3) protein and mitochondrial function using AD human brain samples, demonstrating that dysfunction of SIRT3 leads to mitochondrial and neuronal damage, and may improve the mitochondrial pathology and neurodegeneration in AD (Lee et al., 2017). Therefore, mitochondrial dysfunction is considered as a cardinal pathological hallmark for neurodegenerative diseases including AD and AD-related dementia (Lin and Beal, 2006; van Horssen et al., 2017). Detailed information of mitochondrial activities that may be associated with dementia is presented in Figure 1.

Summarizing the above, the oxidative stress and mitochondrial dysfunction are of high importance in the pathology and pathogenesis of AD and dementia. Therefore, natural antioxidants and mitochondria targeting molecules can be important strategies to treat elderly individuals with AD (Reddy and Reddy, 2017). Several naturally occurring antioxidants, such as *Ginkgo biloba* (Gb) and curcumin, showed blocking effect on the age-dependent spatial cognitive behavior and also increased the Aβ-degrading enzymes in transgenic mouse models (Stackman et al., 2003; Wang et al., 2014). Moreover, several other antioxidants, including ferulic acid, α-lipoic acid, R-lipoic acid, vitamin E, vitamin C, melatonin, CoQ10, *N*-acetyl-L-cysteine, pyrrolyl-alpha-nitronyl nitroxide, and zeolite supplementation, also showed beneficial effects on AD in different transgenic mouse models (Reddy and Reddy, 2017). Most of these naturally occurring compounds revealed reduction in Aβ levels, mitochondrial dysfunction, phosphorylated tau, and microglial activation, and also increased the synaptic activity (Reddy and Reddy, 2017). Therefore, many of them are available as supplements or can be used as alternative treatment strategies that may help certain neurological conditions, such as PD, AD, some dementia types, and other clinical conditions.

## Current pharmaceutical treatments of dementia

### Approved (semi-)synthetic drugs

Several (semi-)synthetic drugs are available worldwide for the treatment of AD and some dementia types. The selective, reversible acetylcholinesterase (AChE) inhibitor donepezil, the non-selective butyrylcholinetserase (BuChE) and AChE inhibitor rivastigmine, as well as the *N*-methyl-D-aspartate (NMDA) receptor antagonist memantine are some of the most widely used therapeutics for AD-associated dementia (Blennow et al., 2006; Winblad et al., 2016). Some of these drugs, like the (semi-)synthetic drug rivastigmine, are also approved for treating other dementia types like PD-related dementia (Winblad et al., 2016). Most prominent drugs approved for clinical use in AD and different forms of dementia are present in Table 2. A combined donepezil–memantine drug with the brand name Namzaric® was approved by the FDA in 2014 for the treatment of moderate-to-severe AD in people who are taking donepezil hydrochloride at the recommended clinically efficient dose of 10 mg/day (http://www.alz.org AD report). However, this combinative medicine may cause various side effects, including muscle problems, slow heartbeat and fainting, increased stomach acid levels, nausea, vomiting, and seizures. In addition, some findings suggest that abovementioned drugs do not provide therapeutic benefits for agitation present in patients with severe behavioral symptoms (Howard et al., 2007; Fox et al., 2012). Some neuroleptic/antipsychotic drugs, such as haloperidol, risperidone, and olanzapine, are currently being used to treat behavioral and psychological symptoms of dementia (BPSD), however, their use is controversial (Ballard and Howard, 2006). Therefore, such drugs are not approved by FDA for the treatment of BPSD. Nevertheless, they are still often prescribed off-label as no better treatment for BPSD exists currently (Ibrahim et al., 2012).

**Table 2:** Approved (semi-)synthetic drugs used for the treatment of dementia.

	**Memantine**	**Rivastigmine**	**Donepezil**
Brand name	Namenda® (USA)	Exelon® (USA, Europe)	Aricept® (USA, Europe)
	Axura® (Europe)		
	Ebixa® (Europe)		
	Memando® (Ger)		
Chemical structure	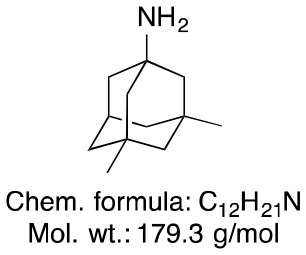	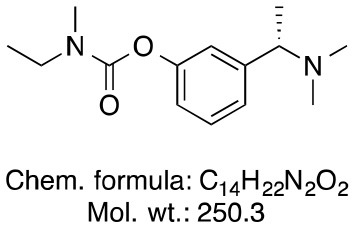	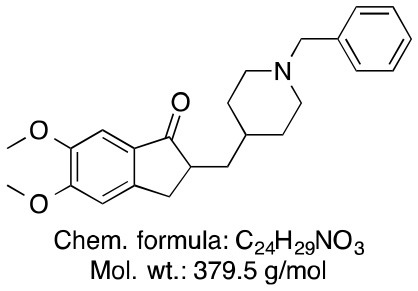
Indications	Moderate-to-severe AD, AD-related dementia	Mild-to-moderate AD, AD-related dementia	Mild-to-moderate AD, early-to-mid AD dementia
Mode of action	Non-competitive NMDA-receptor antagonist	Slowly reversible, non-selective AChE and BuChE inhibitor	Reversible, selective AChE inhibitor
Side effects (http://www.alz.org)	Confusion, dizziness, constipation, and headache	Nausea, vomiting, loss of appetite, increased frequency of bowel movements	Nausea, vomiting, loss of appetite, increased frequency of bowel movements
Half-life (Blennow et al., 2006)	60–100 h (long)	1 h (very short)	70 h (long)
Doses per day (Blennow et al., 2006)	One (first week)	Two	One
Initial dose (Blennow et al., 2006)	5 mg/day	3 mg/day (2 × 1.5 mg)	5 mg/day
Recommended clinically efficient dose (Blennow et al., 2006)	20 mg/day	6–12 mg/day	10 mg/day

The (semi-)synthetic drugs that are currently used for the treatment of dementia have an impact on several symptoms in different disease stages, but do not stop the progressive course of the disease (Tzvetkov and Antonov, 2017). Therefore, the investigation of naturally occurring compounds with potential therapeutic properties for the treatment of different dementia forms is of great medical and socioeconomic importance.

### Galantamine—the only current drug of plant origin against dementia

**Figure 2:**
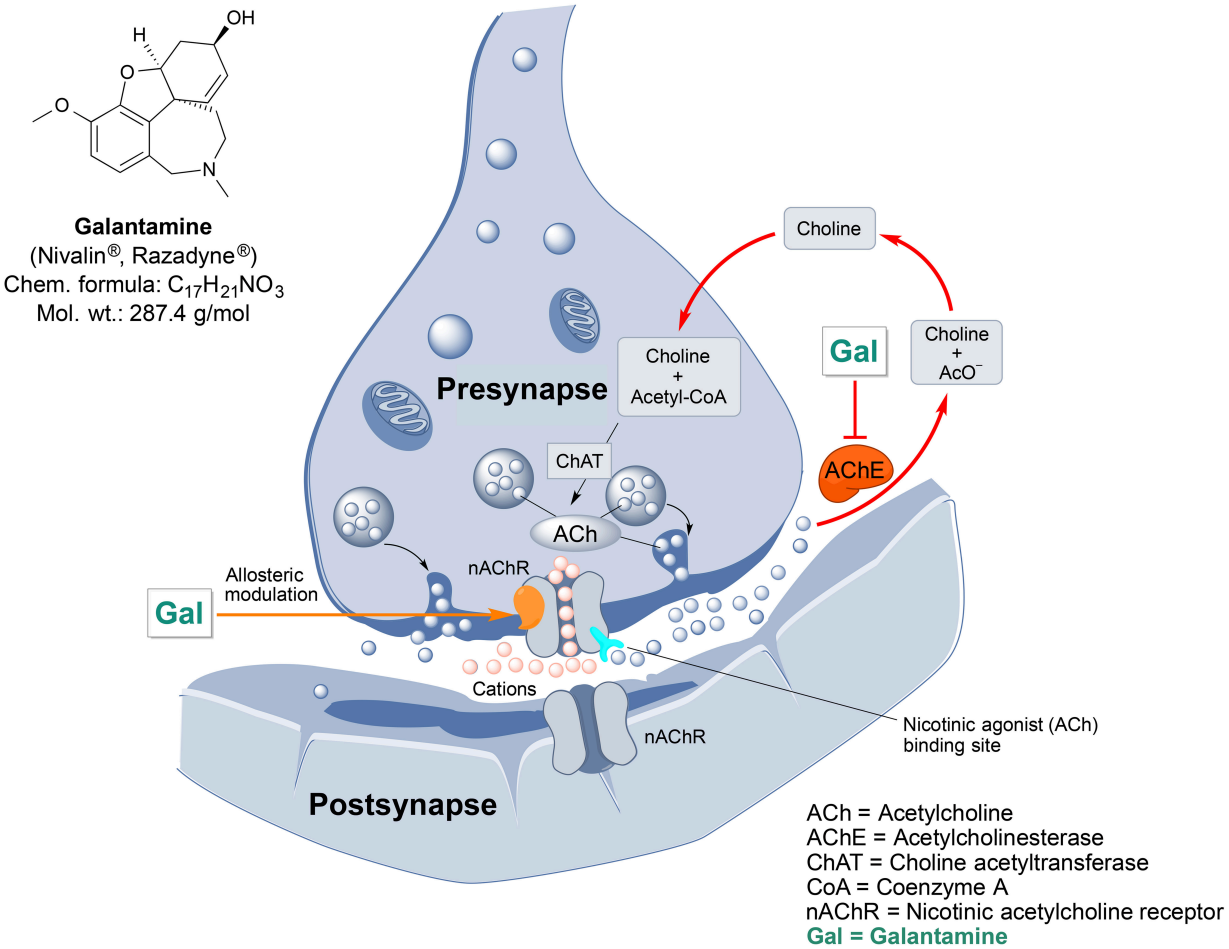
Chemical structure and targeted mechanisms of galantamine (Gal) against AD and dementia. The major biological effects of Gal lead to significant neuroprotection via dual AChE inhibition and allosteric stimulation of nAChRs.

Galantamine is an important drug of plant origin that is widely prescribed for the treatment of mild-to-moderate AD and AD-related dementia (Lilienfeld, 2002). Efficacy of galantamine has been confirmed in several clinical trials (Olin and Schneider, 2002). Galantamine, also known as galanthamine (for structure, see Figure 2), is an isoquinoline alkaloid produced by plants from *Amaryllidaceae* family. It was first discovered and isolated from bulbs of *Galanthus nivalis* (common snowdrop) by the Bulgarian chemist D. Paskov and his team in 1956 (Paskov, 1958). The original industrial phytopreparation of the pure galantamine extract (named Nivalin®) was prepared in late 1950s by the same research group (Chrusciel and Varagić, 1966). Galantamine was first applied to treat poliomyelitis and later, for the treatment of neuropathic pain and as an anesthetic (Ng et al., 2015). Today, galantamine is mainly obtained from *Galanthus woronowi* Losinsk and *Galanthus alpines* Sosn. (Caucasian snowdrop) daffodil bulbs and also synthesized artificially (Loy and Schneider, 2006).

Galantamine has a unique dual mode of action affecting the brain's cholinergic system (Figure 2). It is a reversible, competitive inhibitor of the AChE enzyme and an allosteric enhancer of the nicotinic acetylcholine receptor (nAChR) (Albuquerque et al., 2001).

Galantamine also prevents mitochondrial dysfunction, as shown by its capability to rescue changes in mitochondrial membrane potential (MMP) and morphology induced by Aβ25/35 or hydrogen peroxide treatment (Ezoulin et al., 2008; Liu et al., 2010). Oxidative stress is caused by toxic reactive oxygen species (ROS), which are generated mainly during the electron transport in mitochondria. By protecting the mitochondria and inhibiting AChE activity galantamine decreases oxidative damage to cells and thus mediates neuroprotection (Tsvetkova et al., 2013).

Furthermore, galantamine may interact with other brain-targeted drugs by decreasing activity of P-glycoprotein, a multi-drug resistance transporter present in brain's vascular endothelium (Namanja et al., 2009). It is responsible for actively effluxing drugs back into the bloodstream, thus preventing them from crossing into the brain (Namanja et al., 2009). Hence, galantamine may allow drugs that were co-administered with it to reach the brain more easily. Galantamine also enhances the protective effect of rofecoxib (an anti-inflammatory COX-2 inhibitor) and caffeic acid (a plant-derived phenol) against neurotoxicity-induced mitochondrial dysfunction, oxidative damage, and cognitive impairment in rats (Kumar et al., 2011). Similarly, galantamine potentiates antioxidative activity of melatonin, a brain sleep hormone (Romero et al., 2010). Combined galantamine and memantine treatment is also hypothesized to be a potential novel therapy for schizophrenia (Koola et al., 2014).

Treatment with galantamine has shown to consistently delay the onset of different behavioral symptoms of dementia, such as anxiety, euphoria, depression, irritability, delusions, and unusual motor behavior (Monsch and Giannakopoulos, 2004). A review by Loy and Schneider (2006) described in detail 10 clinical trials comprising the total of 6,805 demented patients, who were submitted to galantamine treatment. The results revealed that the galantamine is well-tolerated by the majority of patients. Some common side effects were observed in the treatment groups in a dose-dependent manner (Loy and Schneider, 2006).

Because of its efficacy, the drug is recommended by Alzheimer's disease/dementia treatment guidelines of the USA and Europe (Doody et al., 2001; Waldemar et al., 2007). The drug is also approved for use in roughly 29 countries including Canada, in the European Union (except for The Netherlands, under the name Nivalin® in 2000), Japan, Korea, Mexico, Singapore, South Africa, Thailand, etc. The FDA approved galantamine in the United States under the brand name Razadyne® in 2001 for the treatment of AD and AD-related dementia. In summary, the plant-derived galantamine is a well-established medicine for dementia treatment, which acts via modulation of acetylcholine signaling and inhibition of oxidative damage.

## General overview of the diversity of plants used in dementia treatment

Modern research on dementias showed that they are complex diseases with multiple molecular mechanisms involved in their pathogenesis. With this realization emerged the new paradigm for treating these pathologies: therapies for dementia should target multiple underling molecular targets, instead of concentrating on any single one. Correspondingly, plant and plant extracts are composed of many substances that are hypothesized to act on multiple molecular targets in an additive or even synergistic manner (Long et al., 2015). Many herbal medicines are already being used for dementia treatment. Unfortunately, active ingredients of these herbs are poorly described. Similarly, we still know very little on how this myriad of substances interact with each other and with prescription medications (Zhou et al., 2016b). The research on these topics will be essential for developing therapeutics, comprised of substances that amplify each other activity and which are devoid of harmful side effects.

In this work, we attempted to collect and document scattered information from various ethnopharmacological reports. We searched several web databases namely, ScienceDirect, Pubmed, Scopus, and Google Scholar using keywords such as “dementia,” “Alzheimer's,” “traditional medicine,” “ethnopharmacology,” and “ethnobotany.” Web hits from Google scholar were gathered through Boolean information retrieval method using plant name with “AND” operator (Pohl et al., 2010) followed by “dementia” or “Alzheimer's.” An overview of the identified medicinal plants used for treating dementia or AD is presented in Table 3.

**Table 3:** Overview of medicinal plants used to treat dementia worldwide.

**Plant (Plant family)**	**References for the use of the plants for dementia treatment in ethnomedicine**	**Number of google scholar hits for keyword “dementia”**	**Number of google scholar hits for keyword “Alzheimer's”**
*Acorus calamus* L. (Acoraceae)	Howes and Houghton, 2003	492	730
*Aframomum melegueta* (Roskoe) K. Schum. (Zingiberaceae)	Fatumbi, 1995	46	104
*Agapanthus africanus* (Agapanthaceae)	Stafford et al., 2008	06	06
*Ammocharis coranica* (Ker-Gawl.) Herb. (Amaryllidaceae)	Stafford et al., 2008	13	26
*Ananas comosus* (Bromeliaceae)	Wolters, 1994; Adams et al., 2007	129	414
*Angelica sinensis* (Oliv.) Diels (Apiaceae)	Mantle et al., 2000	1,010	1,660
*Angelica archangelica* (Apiaceae)	Ross, 2001; Howes and Houghton, 2003	196	318
*Angelica* species (Apiaceae)	Perry and Howes, 2011	4,670	3,140
*Annona senegalensis* Pers. (Annonaceae)	Stafford et al., 2008	139	80
*Artemisia absinthium* L. (Asteraceae)	Howes and Houghton, 2003; Adams et al., 2007	242	439
*Asparagus africanus* Lam. (Asparagaceae)	Stafford et al., 2008	26	34
*Asparagus concinnus* (Baker) Kies (Asparagaceae)	Stafford et al., 2008	01	01
*Bacopa monnieri* (L.) Wettst. (Plantaginaceae)	Manyam, 1999	1,370	1,990
*Barbieria pinnata* (Pers.) Baill. (Fabacea)	Schultes, 1993, 1994; Adams et al., 2007	04	06
*Platycladus orientalis* (L.) Franco (Syn. *Biota orientalis* (L.)Endl.) (Cupressaceae)	Nishiyama et al., 1995; Howes et al., 2003	150	236
*Boophone disticha* (L.f.) Herb. (Amaryllidaceae)	Stafford et al., 2008	39	78
*Brugmansia × candida* Pers. (Solanaceae)	González Ayala, 1994; Adams et al., 2007	12	55
*Bupleurum* species (Apiaceae)	Perry and Howes, 2011	518	717
*Camellia sinensis* Kuntze (Theaceae)	Perry and Howes, 2011	1,420	3,910
*Cannabis sativa* L. (Cannabaceae)	Perry and Howes, 2011	1,740	3,260
*Carum carvi* L. (Apiaceae)	Adsersen et al., 2006	163	340
*Caryophyllus* spp. (Caryophyllaceae)	Tabernaemontanus, 1987; Adams et al., 2007	161	292
*Cassia lucens* Vog. (Fabaceae)	Schultes, 1993, 1994; Adams et al., 2007	28	93
*Celastrus paniculatus* Willd. (Celastraceae)	Howes and Houghton, 2003	189	274
*Centella asiatica* (L.) Urb (Apiaceae)	Stafford et al., 2008	1,060	1,970
*Dysphania ambrosioides* (L.) Mosyakin&Clemants (Amaranthaceae) {Syn. *Chenopodium ambrosioides* L.(Chenopodiaceae)}	de Barradas, 1957; CESA, 1992, 1993; Adams et al., 2007	94	154
*Clitoria ternatea* L. (Fabaceae)	Howes and Houghton, 2003	185	294
*Codonopsis pilulosa* (Franch.) Nannf. (Campanulaceae)	Howes and Houghton, 2003	198	245
*Coffea arabica* L. (Rubiaceae)	Perry and Howes, 2011	484	1,180
*Convallaria majalis* L. (Convallariaceae)	Tabernaemontanus, 1987; Adams et al., 2007	82	95
*Coriandrum sativum* L. (Apiaceae)	Tabernaemontanus, 1987; Adams et al., 2007	310	765
*Corydalis cava* (L.) Schw. et K. (Papaveraceae)	Adsersen et al., 2006; Adams et al., 2007	65	86
*Corydalis intermedia* (L.) M'erat (Papaveraceae)	Adsersen et al., 2006; Adams et al., 2007	71	141
*Corydalis solida* (L.) Swartz ssp. *laxa* (Papaveraceae)	Adsersen et al., 2006; Adams et al., 2007	21	30
*Corydalis solida* (L.) Swartz ssp. *slivenensis* (Papaveraceae)	Adsersen et al., 2006; Adams et al., 2007	04	04
*Crinum bulbispermum* (Burm.f.) Milne-Redh. & Schweick. (Amaryllidaceae)	Stafford et al., 2008	47	97
*Crinum moorei* Hook.f (Syn.*Crinum imbricatum* Baker) (Amaryllidaceae)	Stafford et al., 2008	20	54
*Crinum macowanii* Baker (Amaryllidaceae)	Stafford et al., 2008	41	93
*Crocus sativus* L. (Iridaceae)	Perry and Howes, 2011	678	1,310
*Curcuma longa* L. (Zingiberaceae)	Howes and Houghton, 2003; Perry and Howes, 2011	2,130	6,270
*Euphrasia nemorosa* (Pers.) Wallr. (Scrophulariaceae)	Adsersen et al., 2006	09	16
*Tetradium ruticarpum* (A.Juss.) T.G.Hartley (Syn. *Evodia rutaecarpa* Hook.f.&Thoms.) (Rutaceae)	Mantle et al., 2000; Howes and Perry, 2011	366	367
*Ferula gummosa* Boiss. (Apiaceae)	Tabernaemontanus, 1987; Adams et al., 2007	22	32
*Galanthus woronowii* Losinsk. (Amaryllidaceae)	Perry and Howes, 2011	155	286
*Galanthus alpinus* Sosn. (Syn. *Galanthus caucasicus*) (Amaryllidaceae)	Perry and Howes, 2011	27	55
*Ginkgo biloba* L. (Ginkgoaceae)	Gurib-Fakim, 2006; Perry and Howes, 2011	14,000	17,300
*Glycyrrhiza* species (Leguminosae)	Perry and Howes, 2011	2,410	4,050
*Huperzia serrate* (Thunb.) Trevis. (Lycopodiaceae)	Howes et al., 2003; Adams et al., 2007; Perry and Howes, 2011	1,130	1,710
*Hydrolea glabra* Schum. and Thonn. (Hydrophilaceae)	Fatumbi, 1995; Adams et al., 2007	01	03
*Hypericum perforatum* L. (Clusiaceae)	Perry and Howes, 2011	2,500	3,180
*Lactuca sativa* L. (Asteraceae)	Schweitzer de Palacios, 1994; Adams et al., 2007	287	895
*Lannea schweinfurthii* Engl. (Anacardiaceae)	Stafford et al., 2008	12	22
*Lantana camara* L. (Verbenaceae)	Müller-Ebeling and Rätsch, 1989; Adams et al., 2007	97	262
*Lavandula angustifolia* Miller (Lamiaceae)	Adsersen et al., 2006; Perry and Howes, 2011	800	981
*Leucojum aestivum* L. (Amaryllidaceae)	Perry and Howes, 2011	133	447
*Lycoris radiata* Herb. (Amaryllidaceae)	Howes and Houghton, 2003	227	273
*Magnolia officinalis* Rehder and Wilson (Magnoliaceae)	Howes and Houghton, 2003	389	715
*Matricaria recutita* L. (Asteraceae)	Tabernaemontanus, 1987; Adams et al., 2007	575	1,150
*Medicago sativa* L. (Fabaceae)	Finkler, 1985; Adams et al., 2007	390	1,180
*Melissa officinalis* L. (Lamiaceae)	Lonicerus, 1679; Mills, 1991; Perry et al., 1998; Mantle et al., 2000; Adsersen et al., 2006; Adams et al., 2007; Perry and Howes, 2011	1,140	1,920
*Mentha spicata* L. (Lamiaceae)	Adsersen et al., 2006	223	537
*Narcissus* spp. (Amaryllidaceae)	Perry and Howes, 2011	1,480	745
*Nicotiana* species (Solanaceae)	Perry and Howes, 2011	900	3,060
*Ocimum basilicum* L. (Lamiaceae)	Fuchs, 1543; Sfikas, 1980; Adams et al., 2007	407	997
*Origanum majorana* Moench (Lamiaceae)	Fuchs, 1543; Adams et al., 2007	675	1,550
*Origanum vulgare* L. (Lamiaceae)	Adsersen et al., 2006	358	978
*Paeonia × suffruticosa* Andrews (Paeoniaceae)	Mantle et al., 2000	189	345
*Panax ginseng* C.A.Mey. (Araliaceae)	Perry and Howes, 2011	4,790	6,950
*Paullinia cupana* Kunth ex. H. B. K (Sapindaceae)	Taylor, 1998; Adams et al., 2007	248	515
*Petroselinum crispum* (Mil.) Nym.exA.W.Hill. (Apiaceae)	Adsersen et al., 2006	308	562
*Physostigma venenosa* Balf.f. (Leguminosae)	Mantle et al., 2000	08	11
*Pimpinella anisum* L. (Apiaceae)	Adsersen et al., 2006	214	493
*Piper methysticum* G.Forst. (Piperaceae)	Perry and Howes, 2011	768	953
*Polygala tenuifolia* Willd. (Polygalaceae)	Duke and Ayensu, 1985; Chang et al., 1986; Howes and Houghton, 2003	549	679
*Pteroselinum vulgare* (Mill.) Nym. and A.W. Hill (Apiaceae)	Adams et al., 2007	82	221
*Rosmarinus officinalis* L. (Lamiaceae)	Price and Price, 1995; Chevallier, 1996; Perry et al., 1998; Mantle et al., 2000; Adams et al., 2007	1,140	2,440
*Rosmarinus officinalis* L. (Lamiaceae)	Adsersen et al., 2006	1,140	2,440
*Ruta graveolens* L. (Rutaceae)	Adsersen et al., 2006	351	420
*Salvia lavandulifolia* Vahl. (Lamiaceae)	Perry and Howes, 2011	103	190
*Salvia miltiorrhiza* Bung. (Lamiaceae)	Howes and Houghton, 2003	1,430	2,090
*Salvia officinalis* L. (Lamiaceae)	Sfikas 1980; Tabernaemontanus, 1987; Akhondzadeh et al., 2003; Howes et al., 2003; Savelev et al., 2004; Adams et al., 2007	1,610	2,960
*Scadoxus multiflorus* (Martyn) Raf. (Amaryllidaceae)	Stafford et al., 2008	06	31
*Syzygium aromaticum* (L.) Merrill and Perry (Myrtaceae)	Tabernaemontanus, 1987; Adams et al., 2007	248	647
*Tagetes lucida* Cav. (Asteraceae)	Ortiz de Montellano, 1990; Adams et al., 2007	25	54
*Terminalia chebula* Retz. (Combretaceae)	Misra, 1998; Manyam, 1999; Howes and Houghton, 2003	545	938
*Theobroma cacao* L. (Sterculiaceae)	Roeder, 1988; Adams et al., 2007	535	1,400
*Thymus vulgaris* L. (Lamiaceae)	Adsersen et al., 2006	1,440	2,760
*Valeriana officinalis* L. (Valerianaceae)	Perry and Howes, 2011	973	1,340
*Vinca minor* L. (Apocynaceae)	Perry and Howes, 2011	1,520	2,070
*Vitis vinifera* L. (Vitaceae)	Perry and Howes, 2011	994	3,010

## Selected prominent medicinal plants and plant genera used for the treatment of dementia

After the extensive web-search for medicinal plants used for dementia treatment in various regions worldwide, the following five plants or plant genera described below were selected for detailed discussion according to the highest observed number of web hits.

### *Ginkgo biloba* L.

**Figure 3:**
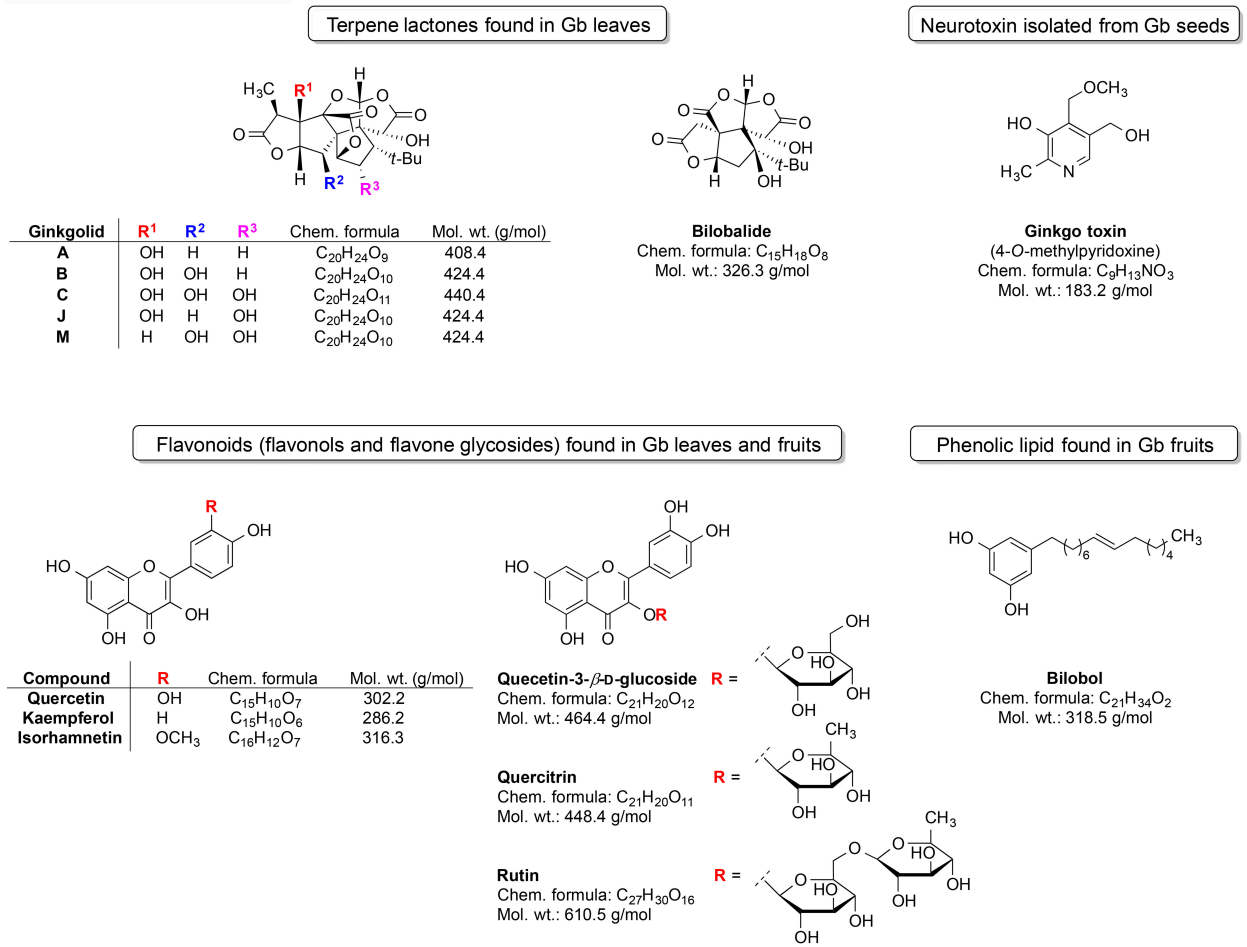
Most prominent phytochemical constituents found in *Gingko biloba* (Gb).

Ginkgo is among the most unique plants on earth and belongs to world's oldest tree species (IARC Working Group, 2016). It is a living fossil which gross morphology did not change for around 200 million years (Guan et al., 2016). Gingko is the last living member of the *Ginkgoaceae* family, which appeared during the Mesozoic era. Gb was cultivated in ancient China due to its diverse medicinal properties. Extracts from this plant were utilized for the treatment of various ailments and symptoms viz. poor circulation, fatigue, vertigo, and tinnitus (Sun et al., 2013). Gingko extract is available on market in some countries (e.g., China) as a herbal supplement named Gingium. It is intended for use in certain age-related cognitive disorders including memory impairment and it alleviates the symptoms of dementias and AD (Zhou et al., 2016a). There are two main categories of chemical phytoconstituents likely responsible for the neuro-therapeutic potential of Gingko: terpene lactones (ginkgolides and bilobalide) and flavonoids (flavonols and flavone glycosides) (Figure 3; Solfrizzi and Panza, 2015; IARC Working Group, 2016).

The triterpene ginkgolides A, B, and C are unique to Gb (Solfrizzi and Panza, 2015). However, there are some other constituents such as the ginkgotoxin (found in Gb seeds) and the phenolic type lipid bilobol (found in Gb fruits) that possess some specific biological effects (IARC Working Group, 2016). For example, ginkgotoxin exhibit neurotoxic activity (induce the epileptic seizures) (IARC Working Group, 2016), whereas bilobol and its derivatives show cytotoxic and antibacterial activity (Tanaka et al., 2011).

**Figure 4:**
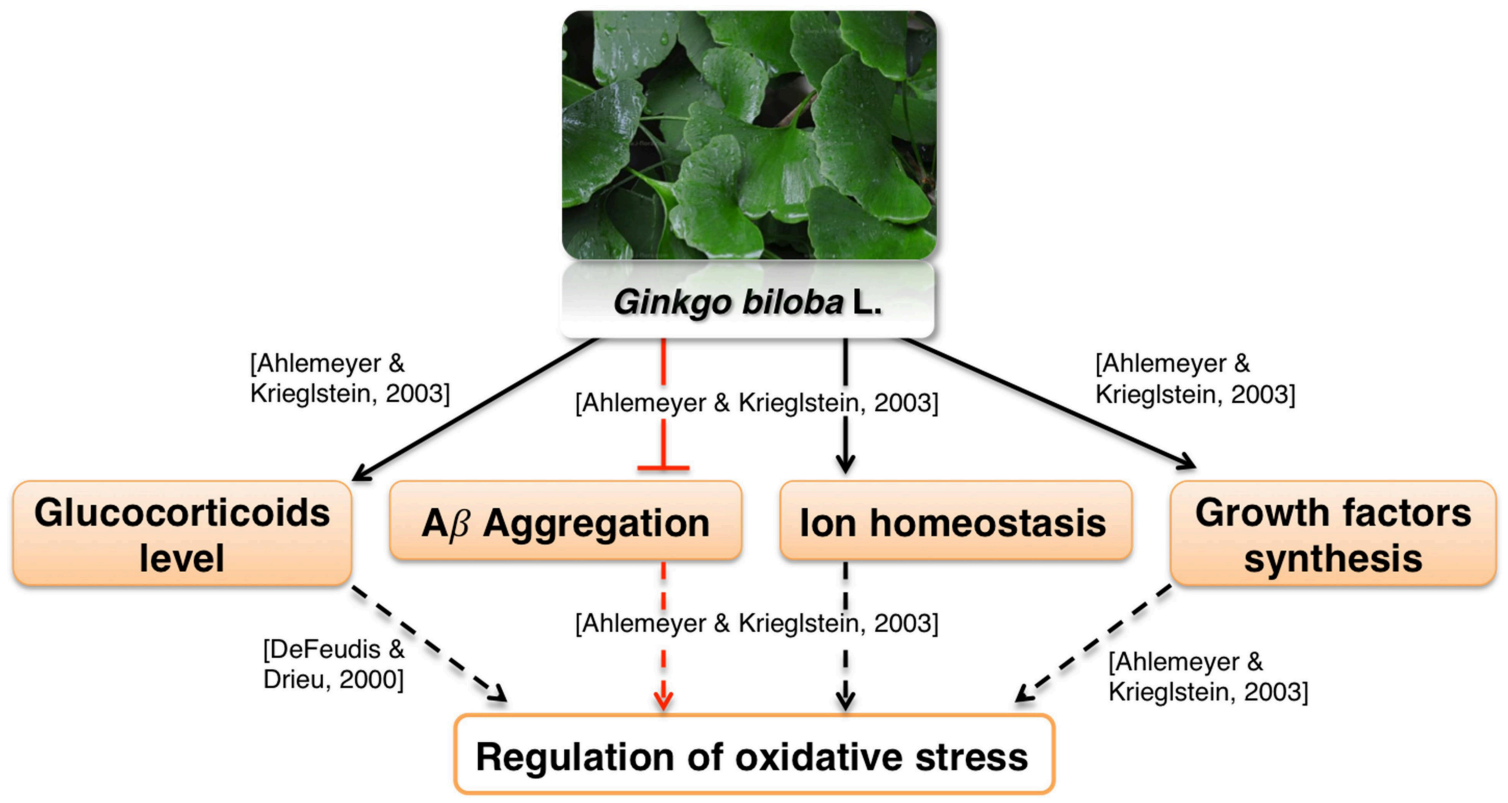
Neuroprotective effects of *Gingko biloba* L.

Ginkgo leaf extract was first developed for therapeutic purposes in Germany in 1965 (Isah, 2015). The first commercially available extract was registered in France in 1974 under the name EGb761; it contains about 24% flavonoids and 6% terpene lactones (Isah, 2015). The standardized Gb extract (EGb761) belongs to the most widely tested in clinical trials herbal medications worldwide for cognitive impairment, AD, and AD-related dementia (Solfrizzi and Panza, 2015). EGb761 affects a multitude of mechanisms associated with proper brain functions (Zhou et al., 2016a; Figure 4).

It was reported to mediate neuroprotection by modulating circulating glucocorticoid levels, as well as Aβ aggregation, ion homeostasis and growth factors synthesis (Amri et al., 1996; Ahlemeyer and Krieglstein, 2003). These processes are likely involved in regulation of oxidative stress (Butterfield et al., 2013; Ruttkay-Nedecky et al., 2013; Dávila et al., 2016; Alam et al., 2017; Gómez-Sámano et al., 2017) which is in agreement with various reports showing antioxidative activity of Gb extract (Bridi et al., 2001; Chandrasekaran et al., 2003). Moreover, the influence of Ginkgo constituents on mitochondrial function is well recognized. Multiple *in vitro* studies show that Ginkgo constituents protect MMP from various toxicants and oxidative stress (Eckert et al., 2005; Abdel-Kader et al., 2007; Wang and Wang, 2016). Gingko extract affects many aspects of mitochondrial morphology such as fission (Zhou et al., 2017), swelling (Schwarzkopf et al., 2013), and coupling (Rhein et al., 2010). Gingko extract also interacts with mitochondrial electron transport chain (Abdel-Kader et al., 2007). Interestingly, it was found that improvement of the oxidative phosphorylation efficiency was more pronounced in cells overexpressing APP than in control cells (Rhein et al., 2010). This suggests that Ginkgo extract may be effective specifically in AD therapy. The extract also protected rodent neurons and glial cells against cerebral ischemia/reperfusion or scopolamine-induced toxicity (Chandrasekaran et al., 2003; Domoráková et al., 2006; Paganelli et al., 2006; Jahanshahi et al., 2012, 2013). Moreover, EGb761 enhanced the functional integrity and protected cerebral microvascular endothelial cells cultured *in vitro* from damage (Yan et al., 2008; Wan et al., 2014). These effects may be related to a known antiplatelet activity of Gingko extract (Kim et al., 2011). Antiplatelet agents are proposed as a possible therapeutics for vascular syndromes (Geeganage et al., 2010; Heim et al., 2017). Thus, EGb761 may counteract dysfunction of neurovascular unit, which is one of the pathologies associated with AD (Farkas and Luiten, 2001; Zlokovic, 2011).

The use of Ginkgo for the treatment of several cerebral dysfunctions connected to neurodegenerative dementia and brain aging has a long history (Abdou et al., 2016). Studies performed on several animal models suggest that Gingko extract may enhance the cognitive and behavioral functions in aged individuals and Parkinson's disease patients (Kim et al., 2004; Takuma et al., 2007; Ribeiro et al., 2016). Apart from the animal studies, several clinical trials also reported the lack of significant adverse effects and effectiveness of EGb 761 in the therapy of AD and vascular dementia (Kanowski et al., 1996; Napryeyenko et al., 2009; Ihl et al., 2012). Although the gingko extract seems to be beneficial for the treatment of cognitive impairments, further studies are required to assess its possible interaction with other drugs. Such studies may translate to better efficacy and safety of gingko extract therapy. Few important interactions between Ginkgo constituents and drugs are currently known (Izzo, 2012). Nevertheless, there are reports of single patients, which suffered serious neurological side effects after administering Ginkgo herb along with risperidone, valproic acid/phenytoin or trazodone (Izzo, 2012). In summary, Ginkgo extract shows neuroprotective effect, which may be underlined by its antioxidative and/or antiplatelet activities. Clinical studies confirm the effectiveness of Ginkgo extract for dementia treatment. Thus, we speculate that some Ginkgo-based drugs may reach the market in near future.

### *Panax ginseng* C.A. Meyer (ginseng)

**Figure 5:**
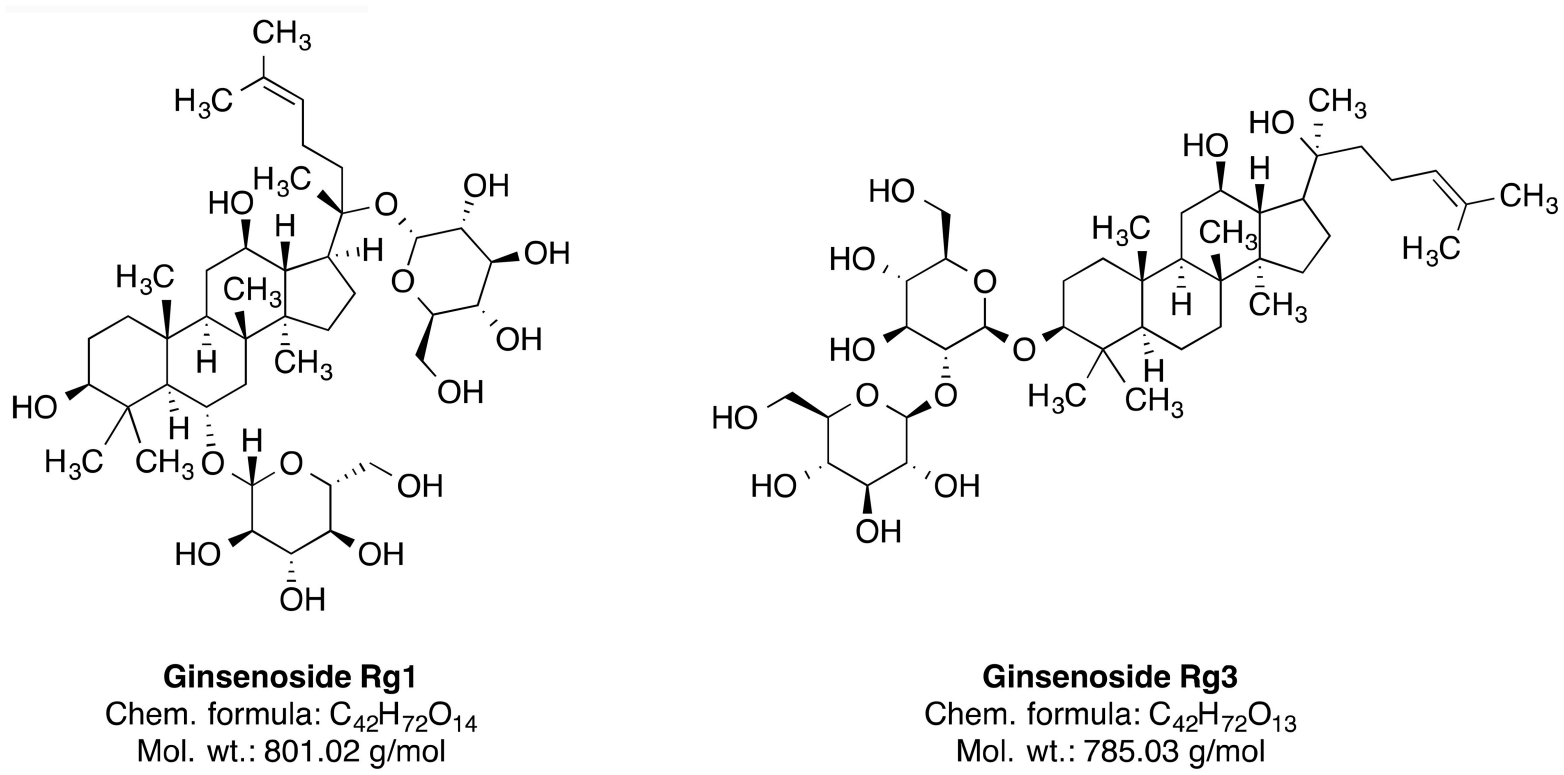
Phytochemical constituents of ginseng.

Ginseng is broadly used as an additive for dietary supplements or medicines. It serves as an adaptogen, which is a substance promoting homeostasis and protecting against various biological stressors. The dried root of this plant was used in the traditional medicine mainly in China and Korea (Yun, 2001). There are several species of *Panax* including *P. ginseng* (Oriental ginseng), *P. japonicus* (Japanese ginseng), *P. quinquefolius* (American ginseng), *P. trifolius, P. notoginseng* (Burkill), and *P. major* (Ngan et al., 1999). *Panax ginseng* CA Meyer is the most frequently used and extensively researched species of ginseng (Lee et al., 2005). The species is widely distributed in the northeastern part of China. The plant has been used in traditional Chinese medicine for a more than 2000 years as a tonic for fatigue, weakness and aging (Wang et al., 2010). Some constituents of this plant, such as ginsenosides Rg1 and Rg3 (Figure 5) and ginseng polysaccharides, have been investigated for their therapeutic potential (Yin et al., 2013; Song et al., 2017; Sun et al., 2017). The neuroprotective effect of ginseng is mainly attributed to the 20(*S*)-ginsenoside Rg3 (Figure 5), which has a steroidal backbone structure with carbohydrate part and aliphatic side chain (Yang et al., 2009). Rg3 is generated by heating the roots at high temperature (Popovich and Kitts, 2004; Sun et al., 2010).

A significant reduction of the amyloid-β40 and amyloid-β42 levels was reported after the ginsenoside Rg3 treatment in the brains of transgenic mice (Tg2576 line), as well as in cultured cells (Chen et al., 2006). Ginsenoside Rg3 also protects against glutamate-induced neurotoxicity in cultured cortical neurons (Kim et al., 1998). Similarly, another ginseng constituent ginsenoside Rg1 (GRg1) suppressed Aβ-induced neurotoxicity, likely through p38 pathway activation in neuroblastoma cells (Li et al., 2012). Ginsenosides also regulate nicotinic acetylcholine receptor channel activity (Nah, 2014). As acetylcholine signaling mediates learning and memory, modulation of acetylcholine receptor activity may be involved in the compound effectiveness against dementia (Bartus et al., 1982; Giacobini, 2004). The ginseng extract specifically enriched with ginsenoside Rg3 rescues scopolamine-induced memory impairment possibly through modulation of the AChE activity and the NF-κB signaling pathway in the hippocampus of mice (Kim et al., 2016). NF-κB is a protein complex, which regulates neuroinflammation and is activated by ROS (Kaur et al., 2015). On a similar note, GRg1 reduces the Aβ-associated generation of ROS and cell death (Wang and Du, 2009). Correspondingly, many publications show protective effect of ginseng constituents on brain mitochondrial activity under multiple toxic conditions, including ischemia (Ye et al., 2011), calcium treatment (Tian et al., 2009; Zhou et al., 2014), hydrogen peroxide treatment (Tian et al., 2009), and even incubation of cells with Aβ *in vitro* (Ma et al., 2014).

A recent meta-analysis of randomized clinical trials showed inconsistent effect of ginseng on AD (Wang et al., 2016). Generally, the trials suffered from small sample size and poor design, including lack of placebo groups (Wang et al., 2016). Thus, there is a need of larger trials to determine the efficacy of ginseng in AD.

In summary, ginseng constituents are suggested to modulate a number of dementia-related mechanisms, such as amyloid-β metabolism, oxidative stress, neuroinflammation, and acetylcholine signaling. Unfortunately, the effect of gingseng on dementia patients remains poorly understood despite some efforts.

### Curcuminoids from genus curcuma

**Figure 6:**
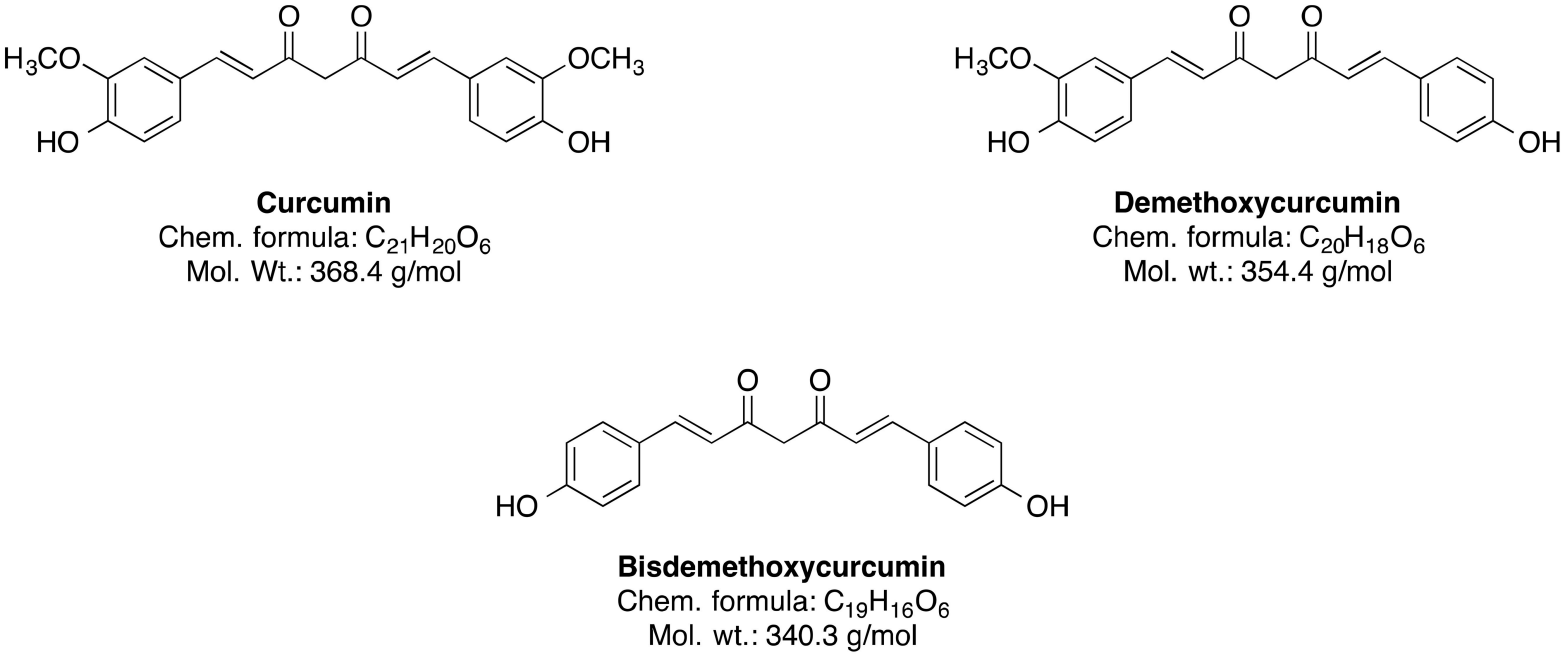
Chemical structures of curcuminoids.

The genus Curcuma (commonly termed as Turmeric) comprises around 80 species and is considered as one of the biggest genera of the *Zingiberaceae* family (Sirirugsa et al., 2007). Curcumin and its curcuminoid analogs, demethoxycurcumin and bisdemethoxycurcumin, are responsible for the typically yellow color of turmeric (Chin et al., 2013). However, the main bioactive phytoconstituent of Curcuma genus is curcumin (Figure 6).

It is an approved natural food colorant (E100) and can be easily obtained from turmeric through solvent extraction and crystallization (Chin et al., 2013). Furthermore, curcumin is widely used in traditional Indian medicine for the treatment of anorexia, hepatic diseases, cold, cough, and other disorders (Chin et al., 2013; Huminiecki et al., 2017). Nowadays, *in vivo* studies suggest that curcumin has potential neuroprotective properties including antioxidant, anti-neuroinflammatory, antiproliferative, anti-amyloidogenic, and neuro-regulative effects (Chin et al., 2013). A large epidemiological Indo-US Cross National Dementia study showed that the peasant Indian population has a low prevalence of AD and AD-associated dementia compared to the US population, and that may be linked to the high curcumin consumption in the Indian population (Chin et al., 2013), although such correlation does not necessarily imply causative connection.

**Figure 7:**
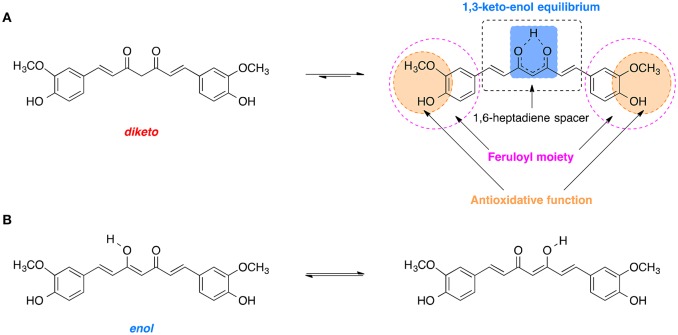
Tautomerism of curcumin: **(A)** Diketo and 1,3-keto-enol equilibrium form of curcumin with its biologically relevant structural units. **(B)** Hydrogen transfer in the most stable enol form.

Curcumin is comprised of two feruloyl moieties with 3-methoxy-4-hydroxy substituents (Figure 7A; Sahne et al., 2017). Both side units are linked together by an unsaturated seven-carbon spacer that includes a β-diketo function so that the molecule of curcumin is almost symmetric. Depending on pH of the environment, curcumin can exist in two possible tautomeric forms: enol and diketo (Sahne et al., 2017). The keto form is predominant in acidic and neutral media (pH ≤ 7.4) as well as in solid state, while in non-polar and basic milieu (pH ≥ 8.0) the enol form is occurring (Sahne et al., 2017). Moreover, the enol form co-exists in two equivalent tautomers that undergo intramolecular hydrogen transfer (Figure 7B; Anjomshoa et al., 2016). Under physiological conditions (pH ~7.4) curcumin can reach its 1,3-keto-enol equilibrium state. Some of the most important antioxidant properties of curcumin and its ability to scavenge ROS are associated with the stability and the antioxidative capability of the methoxy phenolic type groups that are present. The structural features of curcumin including its tautomeric forms and pharmacophores are present in Figure 7.

Curcumin suppresses tumor necrosis factor (TNF) activity, formation of Aβ plaques and protects brain cells from noxious agents (Belkacemi et al., 2011). In recent years, the natural polyphenols are being implemented in the treatment of different neurological disorders (Pathak et al., 2013; Hügel and Jackson, 2015).

**Figure 8:**
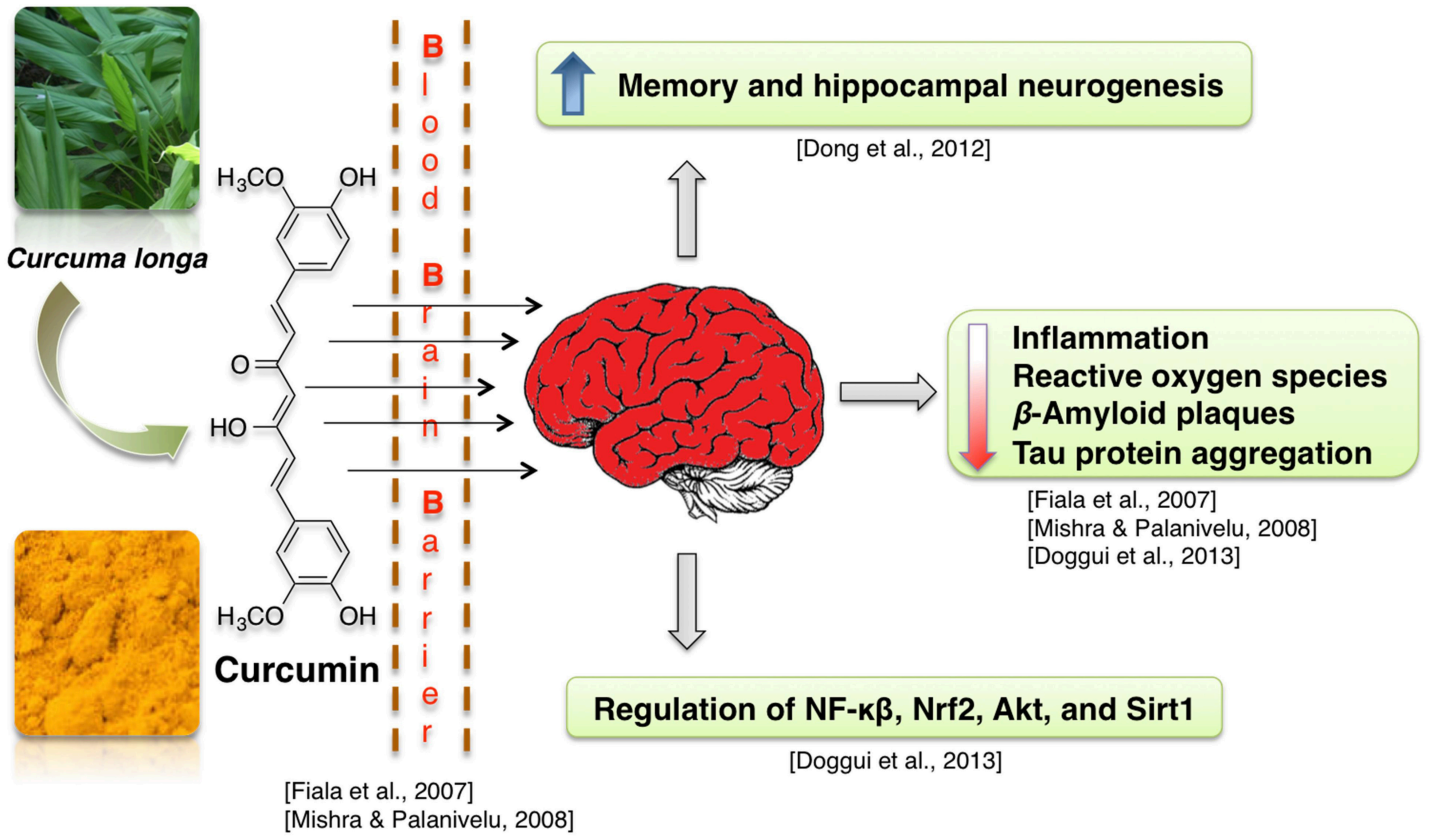
Neuroprotective effects of curcumin.

Curcumin-enriched diet enhances memory and hippocampal neurogenesis in aged rats (Dong et al., 2012). This effect may be mediated by curcumin-induced modulation of expression of genes involved in cell growth and synaptic plasticity (Dong et al., 2012). Neuroprotective properties of curcumin are often attributed to its anti-inflammatory, antioxidant and lipophilic potential (Mishra and Palanivelu, 2008). For example, enhanced hippocampal expression of pro-inflammatory proteins TNF-α and IL-1 beta, which was caused by intracerebroventricular infusion of Aβ42 peptide solution, was at least partially normalized by infusion with curcumin-loaded lipid-core nanocapsules (but not free curcumin) (Hoppe et al., 2013a). Similarly, in neuroblastoma cells, curcumin suppressed the radiation-induced increase in activity of the pro-neuroinflammatory complex NF-κB (Aravindan et al., 2008). Activity of the neuroinflammation- and oxidative damage-related proteins NF-κB, Nrf2, and Sirt1 was also regulated in presence of curcumin in human neuroblastoma cells (Doggui et al., 2013). Curcumin treatment reduces ROS level in neuroblastoma cell lines treated with the noxious agent acrolein and in rat primary neurons with induced Ab42 hyper-expression (Ye and Zhang, 2012; Doggui et al., 2013). Curcumin protects mitochondria from noxious factors such as oxidative stress and rotenone (inhibitor of electron transport chain) *in vitro* (Daverey and Agrawal, 2016; Ramkumar et al., 2017). It also alleviates the age-associated loss of mitochondrial and oxidative activity in rodent brains (Dkhar and Sharma, 2010; Eckert et al., 2013; Rastogi et al., 2014). Curcumin also was shown to stimulate Sirt1 and Bcl-2 expression and to decrease brain cell death in experimental stroke (Miao et al., 2016). Reports also show that curcumin increases cell viability at low dosages (Ye and Zhang, 2012) and binds Aβ peptides, thus preventing them from aggregation into Aβ plaques (Yanagisawa et al., 2011). Due to its lipophillic properties, the curcumin can cross the BBB, bind to the plaques and decrease the β-amyloid plaques in AD by inducing phagocytosis of Aβ (Mishra and Palanivelu, 2008). Some of the curcumin derived pyrazoles and isoxazoles also bind to Aβ42 (Figure 8; Narlawar et al., 2008).

Curcumin treatment decreases Aβ40/42 and PSEN1 protein and mRNA levels in APP-overexpressing neuroblastoma cells (Xiong et al., 2011). Curcumin derivatives also increased the uptake of Aβ by macrophages, which were isolated from blood of AD patients and cultivated *in vitro* (Zhang et al., 2006). This is a relevant information, because peripheral macrophages are known to infiltrate brains of AD patients and participate in clearance of amyloid plaques (Gate et al., 2010). Moreover, in human neuroblastoma cells curcumin inhibits Aβ-induced tau phosphorylation likely via PTEN/Akt/GSK-3β pathway (Huang et al., 2014). Other researchers report, that Akt/GSK-3β and ERK pathways mediate the protective effect of curcumin on Aβ induced memory impairments (Hoppe et al., 2013b; Zhang et al., 2015). Curcumin treatment also improved hippocampal-dependent memory of Aβ-infused rats (Hoppe et al., 2013b).

A systematic review by Brondino et al. showed, that the few clinical trials studying the effect of curcumin on AD yielded inconclusive results (Brondino et al., 2014). Although curcumin was found to be safe during short-term use, future clinical studies need to determine its long-term safety and efficacy on human subjects (Brondino et al., 2014). Since the systematic review arrival, another clinical study was published on the topic, showing improvement of working memory and mood after curcumin treatment in a fairly small group of elderly participants (Cox et al., 2015). There is also a study showing correlation between consumption of curcumin-containing spice—curry—with cognitive performance in elderly (Ng et al., 2006). This finding should be taken with a grain of salt in context of curcumin-dementia research, as curcumin concentration in curry is fairly low (Tayyem et al., 2006) and the extent of biologically available curcumin ingested with curry is disputed. Co-administration of curcumin and donepezil (reversible cholinesterase inhibitor) had synergistic effect on cognition and oxidative stress (Akinyemi et al., 2017). Combined donepezil/curcumin therapeutic also showed good BBB permeability (Yan et al., 2017). Concluding, curcumin protects brain cells against damage induced by oxidative stress and Aβ pathology. These properties may underlie beneficial effects of curcumin for treatment of dementia symptoms, as seen in animal model studies. The data suggest, that curcumin may be a promising candidate for novel dementia medication, but conclusive clinical research that could verify this hypothesis is still lacking. Moreover, to date, researchers analyzed the efficacy of curcumin exclusively against AD-associated dementia, leaving out other dementias.

### *Glycyrrhiza* genus

**Figure 9:**
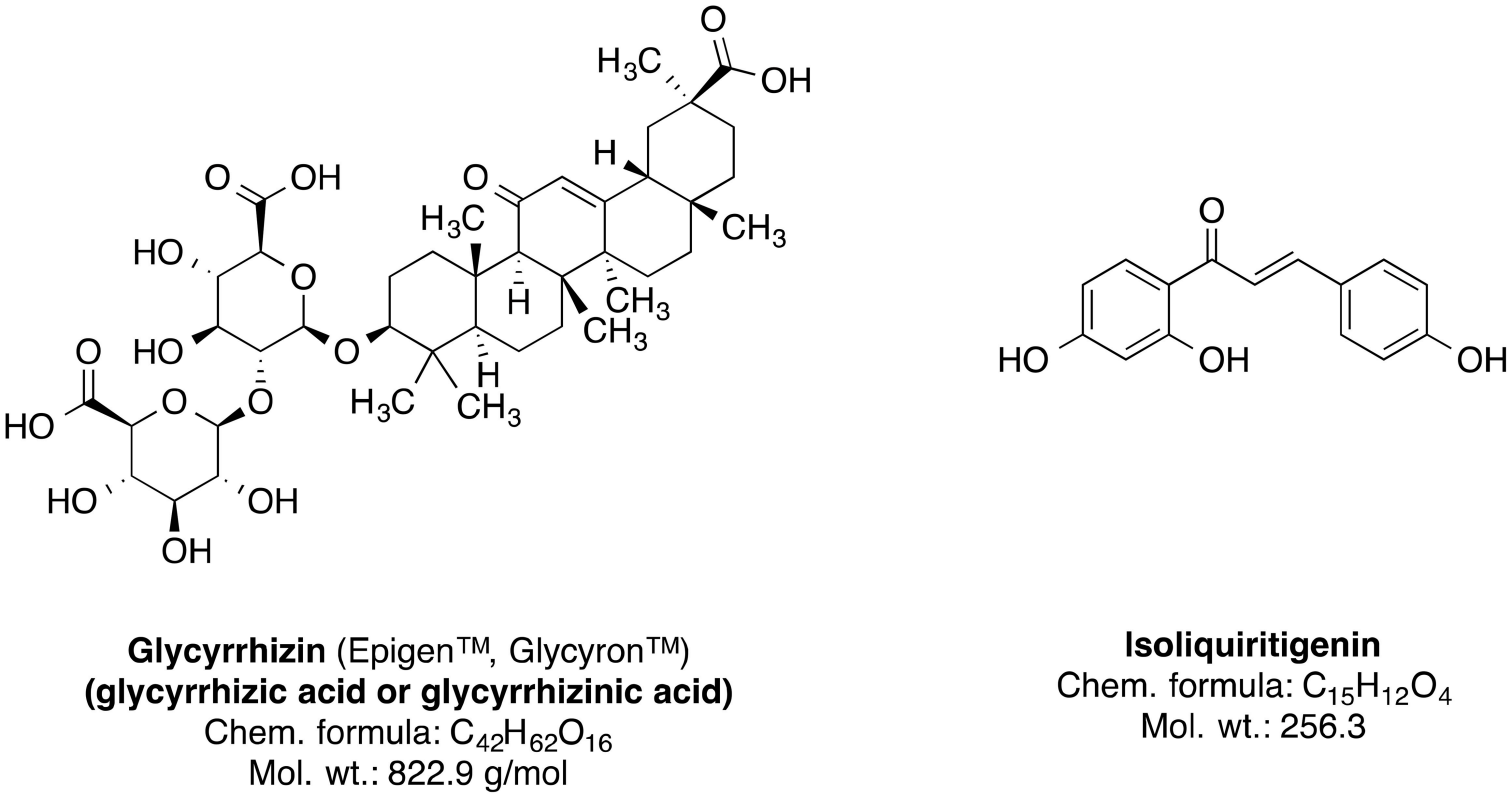
Chemical structures of the major phytoconstituents of *Glycyrrhiza glabra*.

Genus *Glycyrrhiza*, also known as licorice (liquorice), is a member of Fabaceae family and consists of about 30 species. Most of the plants of this genus are perennial herbs native to Mediterranean region, Asia, Southern Russia, and Iran (Asl and Hosseinzadeh, 2008). The *Glycyrrhiza* species are cultivated all throughout Europe and Asia (Blumenthal et al., 2000; Asl and Hosseinzadeh, 2008). The licorice roots and rhizomes are used worldwide as natural sweetener and a herbal medicine mainly for the therapy of autoimmune hepatitis C, jaundice, peptic ulcer, and skin diseases such as atopic dermatitis and inflammation-induced hyperpigmentation (Asl and Hosseinzadeh, 2008; Callender et al., 2011; Tewari et al., 2017); further studies suggest that licorice roots may also have pharmacologically useful properties such as anticancer, antioxidative, anti-inflammatory, antiviral, antimicrobial, hepato- and cardioprotectitve effects (Asl and Hosseinzadeh, 2008; Waltenberger et al., 2016). The main bioactive phytoconstituents of *Glycyrrhiza glabra* (liquorice) root are the sweet-tasting triterpene saponin glycyrrhizin (glycyrrhizic acid) and the phenolic type compound isoliquiritigenin (Figure 9). Other important constituents also include several isoflavonoid derivatives such as shinpterocarpin, glabrone, glabridin, galbrene, lico-isoflavones A and B (Asl and Hosseinzadeh, 2008).

Due to their antioxidative properties, several species of *Glycyrrhiza* were investigated for possible therapeutic effects as neuroprotectants against neurodegenerative disorders such as PD, AD, and dementia. For example, extract from *Glycyrrhiza inflata* prevents tau misfolding *in vitro* (Chang et al., 2016). Thus, the extract of this plant may be effective against various taupathies and AD. *G. inflata* extract decreased oxidative stress in cell models of spinocerebellar ataxia type 3 (SCA3), also known as Machado-Joseph disease (MJD), by upregulating the activity of PPARGC1A and the NFE2L2-ARE pathway (Chen et al., 2014). Glycyrrhizin prevents cytotoxicity, ROS generation and downregulation of glutathione (GSH), which are elicited by 1-methyl-4-phenylpyridinium (MPP^+^) (Yim et al., 2007). MPP+ is a neurotoxic substance acting via interference with the mitochondrial oxidative phosphorylation (Yim et al., 2007). The GSH downregulation is noteworthy, because it is a crucial element of the antioxidative system of the brain (Dringen, 2000). Increased oxidative stress in dementia is attributed to dwindled levels of GSH (Yim et al., 2007; Saharan and Mandal, 2014). Similarly, *G. inflata* extract was shown to inhibit oxidative stress *in vitro* (Chang et al., 2016). The brain cells are susceptible to the oxidative stress. The effect of licorice extract on the oxidative stress may be connected to the beneficial effect of isoliquiritigenin on mitochondrial function (Yang et al., 2012). Licorice may reduce the damage to the brain cells, improve the neuronal function and prevent the memory impairment by diminishing oxidative stress associated with several dementia types (Ju et al., 1989; Dhingra et al., 2004).

**Figure 10:**
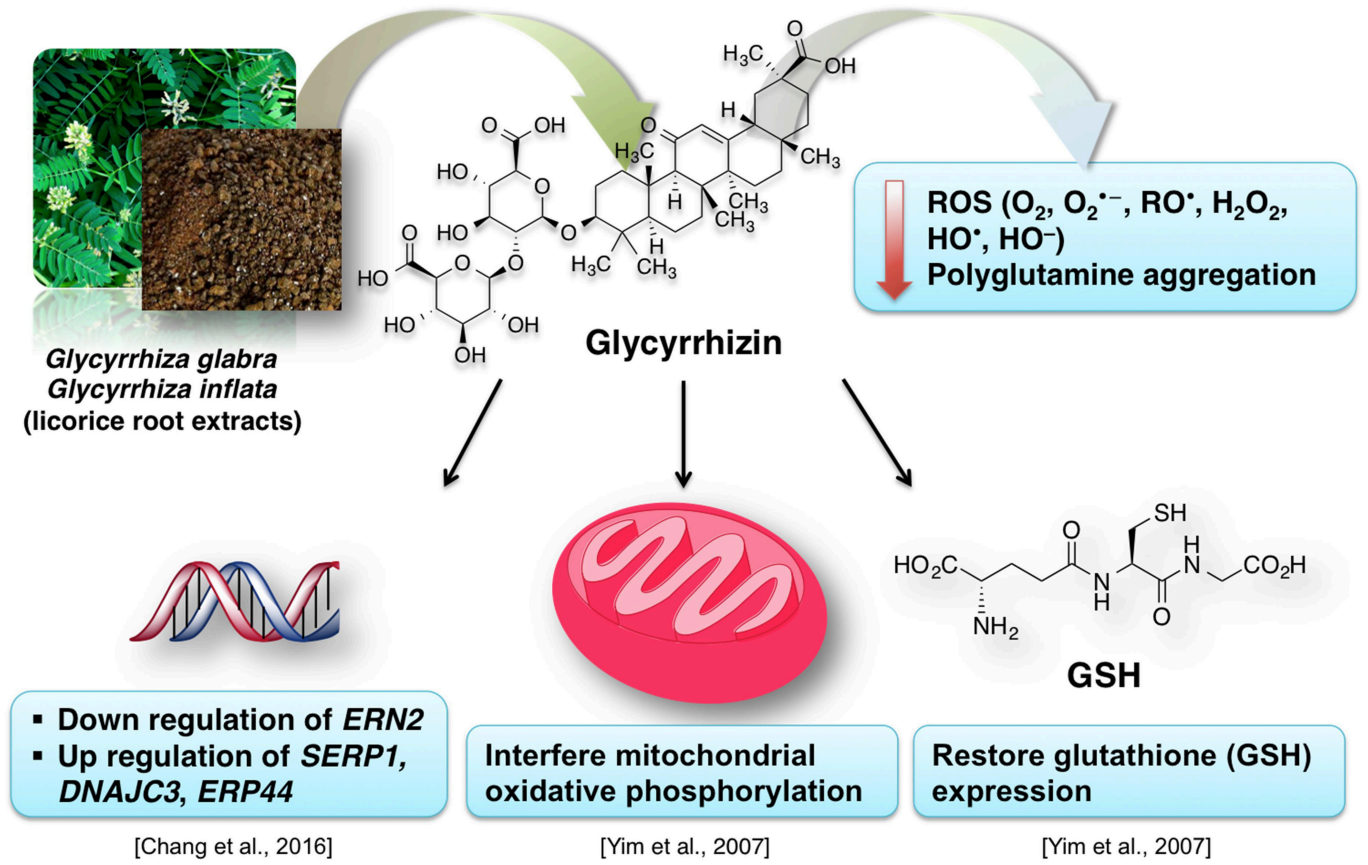
A simplified representation of the neuroprotective effects of licorice for dementia treatment.

Figure 10 summarizes the valuable effects of licorice root extract that may be useful for the treatment of dementia and/or AD-related dementia. Some authors suggest that memory-enhancing activity of the licorice root extract may be connected to its anti-inflammatory effect (Yokota et al., 1998; Dhingra et al., 2004). This data is in agreement with the known tight relationship between inflammation and oxidative stress (Dandekar et al., 2015). *Glycyrrhiza* is used in various polyherbal formulations. One of such formulations used in traditional Japanese Kampo medicine is yokukansan, which is composed of seven different plants including *Glycyrrhiza uralensis* Fisher. *Glycyrrhiza* extract antagonizes α2A adrenoceptors (Ikarashi and Mizoguchi, 2016). Several phytoconstituents of *Glycyrrhiza*: glycyrrhizin, glycycoumarin, liquiritin and isoliquiritigenin show neuroprotective effects when applied as a component of the yokukansan (Ikarashi and Mizoguchi, 2016). Isoliquiritigenin inhibited the activity of the NMDA receptors (Ikarashi and Mizoguchi, 2016). Noteworthy, memantine, an important synthetic drug against dementia, also shows antagonism for NMDA receptors (Danysz and Parsons, 2003). Moreover, the neuroprotective effect of glycycoumarin may be due to its ability to suppress the pro-apoptotic activity of caspase-3 (Kanno et al., 2015; Ikarashi and Mizoguchi, 2016).

Extract from another *Glycyrrhiza* species—*Glycyrrhiza glabra—*improved the learning ability of mice after 7 days of oral administration (Parle et al., 2004). However, another study reported the paradoxical sedative properties of the extract (Hikino, 1985). This shows that the *G. glabra* extract is useful for the improvement of the learning ability but its dose should be established carefully to prevent the sedative effects. A glycyrrhizin salt, diammonium-glycyrrhizinate, prevented the mitochondrial and cognitive dysfunctions induced by Aβ42 in mice (Zhu et al., 2012). Although not yet proven in context of brain function, licorice constituents have a potential to interact with other drugs because of its modulating activity of P450 proteins, which belong to the main regulators of xenobiotic metabolism (Qiao et al., 2014). In summary, glycyrrhiza extracts show anti-inflammatory and antioxidative properties and modulate glutamate signaling and apoptosis. Similarly to above described herbal medicines curcumin and ginseng, despite animal model-based evidence for glycyrrhiza effectiveness in regulating cognitive deficits, there are currently no studies on effectiveness of this plant for dementia therapy in patients.

### *Camellia sinensis* Kuntze

*Camellia sinensis* Kuntze (green tea) brew is one of the most extensively consumed beverages in the world (Goenka et al., 2013). Several beneficial effects of green tea consumption are reported for various conditions like obesity, diabetes, inflammation, coronary artery disease, stroke and some malignancies (de Mejia et al., 2009; Chacko et al., 2010). Consumption of green tea-related compounds [e.g., (-)-epigallocatechin-3-gallate] improves cognitive functions and prevents memory impairment in animals and humans (Rezai-Zadeh et al., 2008; de Mejia et al., 2009; Mandel et al., 2011).

**Figure 11:**
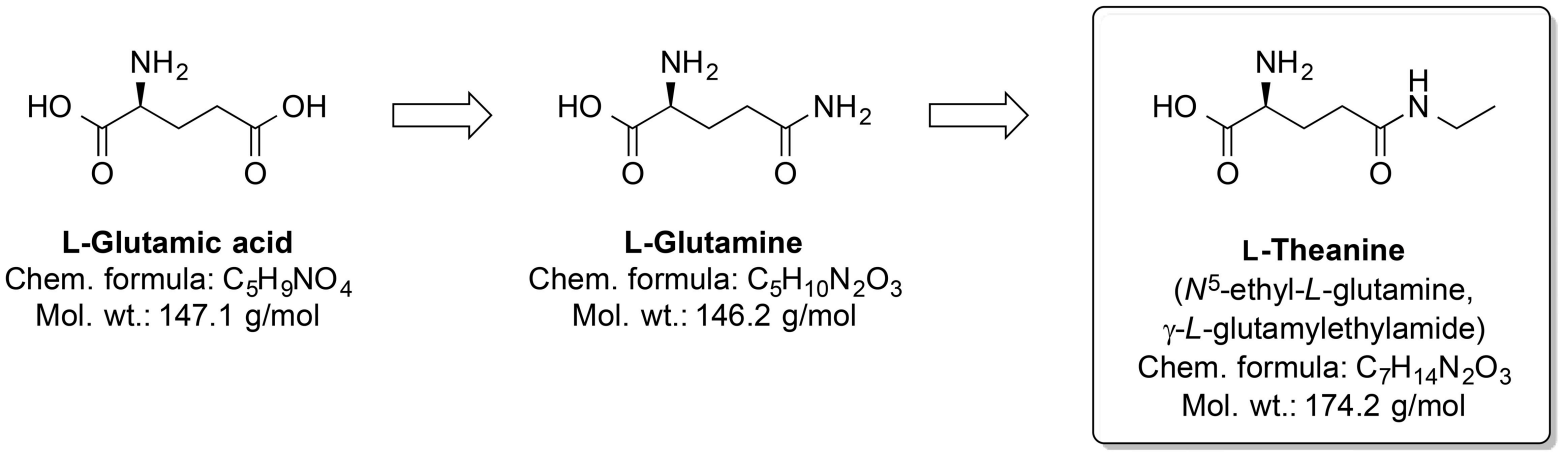
Chemical structures of the nootropic drug L-Theanine and its proteinogenic amino acid analogs.

Around one-third out of 4,000 bioactive compounds of *C. sinensis* are polyphenols (Mahmood et al., 2010). The gene expression and activity of the membrane metalloendopeptidase (MME) were enhanced by green tea extract. Several MME are capable of degrading Aβ peptides (Wang et al., 2006). Pretreatment by infusion with extract of green tea leaves into the left hippocampus of a rat prevented cognitive impairments and superoxide dismutase activity changes, and corrected deregulated activity of pro-inflammatory enzyme COX and AChE, which were induced by injecting AlCl_3_ into the same brain area (Jelenkovic et al., 2014). When injected i.p. L-theanine, one of the amino acid components of *C. sinensis*, protects against memory impairment and cell death caused by ischemia (Egashira et al., 2007, 2008). Interestingly, chronic ingestion of L-theanine in rats also caused improvement in cognitive functions (Yamada et al., 2008) and decreased oxidation levels in the brain of the rats (Nishida et al., 2008). Similarly, in transgenic mouse AD model, the green tea constituent epigallocatechin-3-gallate normalized the dysregulated ROS production, as well as mitochondrial respiration and MMP (Dragicevic et al., 2011). Generally, L-theanine is an *N*-amino-ethylated analog of the proteinogenic neurotransmitter L-glutamic acid and its precursor L-glutamine (Figure 11). L-theanine protects against Aβ42-induced memory deficits and death of cortical and hippocampal cells, possibly by suppressing the ERK/p38 and NF-κB signaling pathways and reducing the oxidative damage (Kim et al., 2009).

Daily consumption of green tea is hypothesized to reduce the risk of AD and age-related dementia (Ayoub and Melzig, 2006). In some clinical studies, L-theanine improved the cognitive functions and mood in combination with caffeine in healthy human subjects (Haskell et al., 2008; Owen et al., 2008). On the other hand, the results of studies on the effects of L-theanine alone on mood are inconclusive (Kimura et al., 2007; Haskell et al., 2008). Green tea catechins can regulate activity of P-glycoprotein (Zhou et al., 2004), which may influence brain availability of co-administered substances. Summarizing, green tea extract shows antiapoptotic and antioxidative activities and may even directly inhibit Aβ plaque formation. Moreover, several human studies provide credibility to the hypothesis that green tea constituents may be effective for modulation of human cognition and perhaps in dementia treatment.

## Conclusions

In this work, we discuss that a large number of plants have been used for dementia treatment worldwide. The mechanisms of action of the reviewed five prominent representative plants generally involve anti-inflammatory, antioxidative, and antiapoptotic activity that are mainly associated with the neuroprotective effects of these plants or their bioactive constituents. Some of such naturally occurring compounds exhibit promising potential as alternative therapeutic strategies. For example, curcumin showed remarkable synergetic effects on cognition and oxidative stress, as well as good BBB permeability, when co-administrated with the approved reversible ChE inhibitor donepezil. Moreover, such combined donepezil/curcumin application may be useful for treatment of dementia symptoms, as seen in animal model studies. Furthermore, combined supplement treatment with some mitochondrial antioxidants, such as α-lipoic acid and acetyl-L-carnitine has been shown to reduce both physical and mental weariness or even to restore memory function in age-related diseases, including AD and different types of dementia, as provided in transgenic mouse model of AD. However, there are still many unknowns in this research area that need to be clarified by planning future studies. The scientific community needs more comprehensive research focused on identifying active ingredients of plants and investigating their mechanism of action. Such research will facilitate clinical studies evaluating the potential of the featured herbal products in dementia treatment. We conclude that further research on plants used in different ethnomedicinal practices and the traditional medicine could lead to the development of novel therapeutics for dementia and, therefore, is of a very high interest.

## Author contributions

DT, AMS, and AA have written the first draft of the manuscript. AM, ANS, LH, JH, and NT revised and improved the first draft. All authors have seen and agreed on the finally submitted version of the manuscript.

### Conflict of interest statement

Author NT was employed by NTZ Lab ltd. The other authors declare that the research was conducted in the absence of any commercial or financial relationships that could be construed as a potential conflict of interest.

## References

[B1] Abbott A. (2011). Dementia: a problem for our age. Nature 475, S2–S4. 10.1038/475S2a 21760579

[B2] Abdel-Kader R. Hauptmann S. Keil U. Scherping I. Leuner K. Eckert A.. (2007). Stabilization of mitochondrial function by *Ginkgo biloba* extract (EGb 761). Pharmacol. Res. 56, 493–502. 10.1016/j.phrs.2007.09.011 17977008

[B3] Abdou H. M. Yousef M. I. El Mekkawy D. A. Al-Shami A. S. (2016). Prophylactic neuroprotective efficiency of co-administration of *Ginkgo biloba* and Trifolium pretense against sodium arsenite-induced neurotoxicity and dementia in different regions of brain and spinal cord of rats. Food Chem. Toxicol. 94, 112–127. 10.1016/j.fct.2016.05.015 27234133

[B4] Adams M. Gmünder F. Hamburger M. (2007). Plants traditionally used in age related brain disorders—a survey of ethnobotanical literature. J. Ethnopharmacol. 113, 363–381. 10.1016/j.jep.2007.07.016 17720341

[B5] Adsersen A. Gauguin B. Gudiksen L. Jäger A. K. (2006). Screening of plants used in Danish folk medicine to treat memory dysfunction for acetylcholinesterase inhibitory activity. J. Ethnopharmacol. 104, 418–422. 10.1016/j.jep.2005.09.032 16280217

[B6] Ahlemeyer B. Krieglstein J. (2003). Neuroprotective effects of *Ginkgo biloba* extract. Cell. Mol. Life Sci. C. 60, 1779–1792. 10.1007/s00018-003-3080-1 14523543 PMC11146048

[B7] Akhondzadeh S. Noroozian M. Mohammadi M. Ohadinia S. Jamshidi A. H. Khani M. (2003). Salvia officinalis extract in the treatment of patients with mild to moderate Alzheimer's disease: a double blind, randomized and placebo-controlled trial. J. Clin. Pharm. Ther. 28, 53–59. 10.1046/j.1365-2710.2003.00463.x 12605619

[B8] Akinyemi A. J. Oboh G. Oyeleye S. I. Ogunsuyi O. (2017). Anti-amnestic effect of curcumin in combination with donepezil, an anticholinesterase drug: involvement of cholinergic system. Neurotox. Res. 31, 560–569. 10.1007/s12640-017-9701-5 28102474

[B9] Alam M. M. Okazaki K. Nguyen L. T. T. Ota N. Kitamura H. Murakami S.. (2017). Glucocorticoid receptor signaling represses the antioxidant response by inhibiting histone acetylation mediated by the transcriptional activator NRF2. J. Biol. Chem. 292, 7519–7530. 10.1074/jbc.M116.773960 28314773 PMC5418050

[B10] Albuquerque E. X. Santos M. D. Alkondon M. Pereira E. F. Maelicke A. (2001). Modulation of nicotinic receptor activity in the central nervous system: a novel approach to the treatment of Alzheimer disease. Alzheimer Dis. Assoc. Disord. 15(Suppl. 1), S19–25. 10.1097/00002093-200108001-00004 11669505

[B11] Aminzadeh M. Roghani M. Sarfallah A. Riazi G. H. (2018). TRPM2 dependence of ROS-induced NLRP3 activation in Alzheimer's disease. Int. Immunopharmacol. 54, 78–85. 10.1016/j.intimp.2017.10.024 29107864

[B12] Amri H. Ogwuegbu S. O. Boujrad N. Drieu K. Papadopoulos V. (1996). *In vivo* regulation of peripheral-type benzodiazepine receptor and glucocorticoid synthesis by *Ginkgo biloba* extract EGb 761 and isolated ginkgolides. Endocrinology 137, 5707–5718. 10.1210/endo.137.12.8940403 8940403

[B13] Anjomshoa S. Namazian M. Noorbala M. R. (2016). The effect of solvent on tautomerism, acidity and radical stability of curcumin and its derivatives based on thermodynamic quantities. J. Solution Chem. 45, 1021–1030. 10.1007/s10953-016-0481-y

[B14] Antico Arciuch V. G. Elguero M. E. Poderoso J. J. Carreras M. C. (2012). Mitochondrial regulation of cell cycle and proliferation. Antioxid. Redox Signal. 16, 1150–1180. 10.1089/ars.2011.4085 21967640 PMC3315176

[B15] Aravindan N. Madhusoodhanan R. Ahmad S. Johnson D. Herman T. S. (2008). Curcumin inhibits NFκB mediated radioprotection and modulate apoptosis related genes in human neuroblastoma cells. Cancer Biol. Ther. 7, 569–576. 10.4161/cbt.7.4.5534 18305409

[B16] Asl M. N. Hosseinzadeh H. (2008). Review of pharmacological effects of *Glycyrrhiza* sp. and its bioactive compounds. Phyther. Res. 22, 709–724. 10.1002/ptr.2362 18446848 PMC7167813

[B17] Atanasov A. G. Waltenberger B. Pferschy-Wenzig E.-M. Linder T. Wawrosch C. Uhrin P.. (2015). Discovery and resupply of pharmacologically active plant-derived natural products: a review. Biotechnol. Adv. 33, 1582–1614. 10.1016/j.biotechadv.2015.08.001 26281720 PMC4748402

[B18] Ayoub S. Melzig M. F. (2006). Induction of neutral endopeptidase (NEP) activity of SK-N-SH cells by natural compounds from green tea. J. Pharm. Pharmacol. 58, 495–501. 10.1211/jpp.58.4.0009 16597367

[B19] Ballard C. Howard R. (2006). Neuroleptic drugs in dementia: benefits and harm. Nat. Rev. Neurosci. 7, 492–500. 10.1038/nrn1926 16715057

[B20] Baloh R. H. (2011). TDP-43: the relationship between protein aggregation and neurodegeneration in amyotrophic lateral sclerosis and frontotemporal lobar degeneration. FEBS J. 278, 3539–3549. 10.1111/j.1742-4658.2011.08256.x 21777387 PMC3177991

[B21] Banasch M. Ellrichmann M. Tannapfel A. Schmidt W. E. Goetze O. (2011). The non-invasive (13)C-methionine breath test detects hepatic mitochondrial dysfunction as a marker of disease activity in non-alcoholic steatohepatitis. Eur. J. Med. Res. 16:258. 10.1186/2047-783X-16-6-258 21810560 PMC3353401

[B22] Bartus R. T. Dean R. L. III. Beer B. Lippa A. S. (1982). The cholinergic hypothesis of geriatric memory dysfunction. Science 217, 408–414. 10.1126/science.7046051 7046051

[B23] Beal M. F. (2005). Mitochondria take center stage in aging and neurodegeneration. Ann. Neurol. 58, 495–505. 10.1002/ana.20624 16178023

[B24] Belkacemi A. Doggui S. Dao L. Ramassamy C. (2011). Challenges associated with curcumin therapy in Alzheimer disease. Expert Rev. Mol. Med. 13, e34. 10.1017/S1462399411002055 22051121

[B25] Bennett S. Grant M. M. Aldred S. (2009). Oxidative stress in vascular dementia and Alzheimer's disease: a common pathology. J. Alzheimers. Dis. 17, 245–257. 10.3233/JAD-2009-1041 19221412

[B26] Berridge M. J. (1995). Calcium signalling and cell proliferation. Bioessays 17, 491–500. 10.1002/bies.950170605 7575490

[B27] Berridge M. J. (2011). Calcium signalling and Alzheimer's disease. Neurochem. Res. 36, 1149–1156. 10.1007/s11064-010-0371-4 21184278

[B28] Blennow K. de Leon M. J. Zetterberg H. (2006). Alzheimer's disease. Lancet (London, England) 368, 387–403. 10.1016/S0140-6736(06)69113-7 16876668

[B29] Blumenthal M. Goldberg A. Brinkmann J. (2000). Herbal Medicine, Expanded Commission E Monographs Cd-Rom. Austin, TX: American Botanical Council/Integrative Medical.

[B30] Bridi R. Crossetti F. P. Steffen V. M. Henriques A. T. (2001). The antioxidant activity of standardized extract of *Ginkgo biloba* (EGb 761) in rats. Phytother. Res. 15, 449–451. 10.1002/ptr.814 11507743

[B31] Brondino N. Re S. Boldrini A. Cuccomarino A. Lanati N. Barale F.. (2014). Curcumin as a therapeutic agent in dementia: a mini systematic review of human studies. Sci. World J. 2014:174282. 10.1155/2014/174282 24578620 PMC3919104

[B32] Brun A. (1987). Frontal lobe degeneration of non-Alzheimer type. I. Neuropathology. Arch. Gerontol. Geriatr. 6, 193–208. 10.1016/0167-4943(87)90021-5 3689053

[B33] Burgess L. Page S. Hardman P. (2002). Changing attitudes in dementia care and the role of nurses. Nurs. Times 99, 18–19. 14562428

[B34] Burte F. Carelli V. Chinnery P. F. Yu-Wai-Man P. (2015). Disturbed mitochondrial dynamics and neurodegenerative disorders. Nat. Rev. Neurol. 11, 11–24. 10.1038/nrneurol.2014.228 25486875

[B35] Butterfield D. A. Swomley A. M. Sultana R. (2013). Amyloid beta-peptide (1-42)-induced oxidative stress in Alzheimer disease: importance in disease pathogenesis and progression. Antioxid. Redox Signal. 19, 823–835. 10.1089/ars.2012.5027 23249141 PMC3749710

[B36] Cai H. Cong W. Ji S. Rothman S. Maudsley S. Martin B. (2012). Metabolic dysfunction in Alzheimer's disease and related neurodegenerative disorders. Curr. Alzheimer Res. 9, 5–17. 10.2174/156720512799015064 22329649 PMC4097094

[B37] Callender V. D. St Surin-Lord S. Davis E. C. Maclin M. (2011). Postinflammatory hyperpigmentation: etiologic and therapeutic considerations. Am. J. Clin. Dermatol. 12, 87–99. 10.2165/11536930-000000000-00000 21348540

[B38] CESA (1992). Usos Tradicionales de las Especies Forestales Nativas en el Ecuador. Tomo II. CESA: Central Ecuatoriana de Servicios Agrícolas.

[B39] CESA (1993). Usos Tradicionales de las Especies Forestales Nativas en el Ecuador. Tomo III. CESA: Central Ecuatoriana de Servicios Agrícolas.

[B40] Chacko S. M. Thambi P. T. Kuttan R. Nishigaki I. (2010). Beneficial effects of green tea: a literature review. Chin. Med. 5:13. 10.1186/1749-8546-5-13 20370896 PMC2855614

[B41] Chan D. C. (2006). Mitochondria: dynamic organelles in disease, aging, and development. Cell 125, 1241–1252. 10.1016/j.cell.2006.06.010 16814712

[B42] Chandrasekaran K. Mehrabian Z. Spinnewyn B. Chinopoulos C. Drieu K. Fiskum G. (2003). Neuroprotective effects of bilobalide, a component of *Ginkgo biloba* extract (EGb 761®) in global brain ischemia and in excitotoxicity-induced neuronal death. Pharmacopsychiatry 36, 89–94. 10.1055/s-2003-40447 13130395

[B43] Chang H.-M. But P. P. H. Yao S.-C. (1986). Pharmacology and Applications of Chinese Materia Medica. Singapore: World Scientific.

[B44] Chang K.-H. Chen I.-C. Lin H.-Y. Chen H.-C. Lin C.-H. Lin T.-H.. (2016). The aqueous extract of Glycyrrhiza inflata can upregulate unfolded protein response-mediated chaperones to reduce tau misfolding in cell models of Alzheimer's disease. Drug Des. Devel. Ther. 10, 885–896. 10.2147/DDDT.S96454 27013866 PMC4778784

[B45] Chen C.-M. Weng Y.-T. Chen W.-L. Lin T.-H. Chao C.-Y. Lin C.-H.. (2014). Aqueous extract of Glycyrrhiza inflata inhibits aggregation by upregulating PPARGC1A and NFE2L2-ARE pathways in cell models of spinocerebellar ataxia 3. Free Radic. Biol. Med. 71, 339–350. 10.1016/j.freeradbiomed.2014.03.023 24675225

[B46] Chen F. Eckman E. A. Eckman C. B. (2006). Reductions in levels of the Alzheimer's amyloid beta peptide after oral administration of ginsenosides. FASEB J. Off. Publ. Fed. Am. Soc. Exp. Biol. 20, 1269–1271. 10.1096/fj.05-5530fje 16636099

[B47] Chen J. X. Yan S. S. (2010). Role of mitochondrial amyloid-beta in Alzheimer's disease. J. Alzheimers. Dis. 20(Suppl. 2), S569–S578. 10.3233/JAD-2010-100357 20463403

[B48] Chevallier A. (1996). The Encyclopedia of Medicinal Plants. London: Dorling Kindersley publishers.

[B49] Chin D. Huebbe P. Pallauf K. Rimbach G. (2013). Neuroprotective properties of curcumin in Alzheimer's disease–merits and limitations. Curr. Med. Chem. 20, 3955–3985. 10.2174/09298673113209990210 23931272

[B50] Chrusciel M. Varagić V. (1966). The effect of galantamine on the blood pressure of the rat. Br. J. Pharmacol. 26, 295–301. 10.1111/j.1476-5381.1966.tb01908.x 4380412 PMC1510659

[B51] Corder E. H. Saunders A. M. Strittmatter W. J. Schmechel D. E. Gaskell P. C. Small G. W.. (1993). Gene dose of apolipoprotein E type 4 allele and the risk of Alzheimer's disease in late onset families. Science 261, 921–923. 8346443 10.1126/science.8346443

[B52] Corrada M. M. Brookmeyer R. Paganini-Hill A. Berlau D. Kawas C. H. (2010). Dementia incidence continues to increase with age in the oldest old: the 90+ study. Ann. Neurol. 67, 114–121. 10.1002/ana.21915 20186856 PMC3385995

[B53] Cox K. H. M. Pipingas A. Scholey A. B. (2015). Investigation of the effects of solid lipid curcumin on cognition and mood in a healthy older population. J. Psychopharmacol. 29, 642–651. 10.1177/0269881114552744 25277322

[B54] Cummings J. Aisen P. S. DuBois B. Frölich L. Jack C. R. Jones R. W.. (2016). Drug development in Alzheimer's disease: the path to 2025. Alzheimer's Res. Ther. 8:39. 10.1186/s13195-016-0207-9 27646601 PMC5028936

[B55] Dai D.-F. Chiao Y. A. Marcinek D. J. Szeto H. H. Rabinovitch P. S. (2014). Mitochondrial oxidative stress in aging and healthspan. Longev. Heal. 3:6. 10.1186/2046-2395-3-6 24860647 PMC4013820

[B56] Damasio A. R. Gabrowski T. J. (2004). Definition, clinical features and neuroanatomical basis of dementia, in The Neuropathology of Dementia, eds Esiri M. M. Lee V. M.-Y. Trojanowski J. Q. (Cambridge: Cambridge University Press), 1–34.

[B57] Dandekar A. Mendez R. Zhang K. (2015). Cross talk between ER stress, oxidative stress, and inflammation in health and disease. Methods Mol. Biol. 1292, 205–214. 10.1007/978-1-4939-2522-3_15 25804758

[B58] Danysz W. Parsons C. G. (2003). The NMDA receptor antagonist memantine as a symptomatological and neuroprotective treatment for Alzheimer's disease: preclinical evidence. Int. J. Geriatr. Psychiatry 18, S23–S32. 10.1002/gps.938 12973747

[B59] Daverey A. Agrawal S. K. (2016). Curcumin alleviates oxidative stress and mitochondrial dysfunction in astrocytes. Neuroscience 333, 92–103. 10.1016/j.neuroscience.2016.07.012 27423629

[B60] Dávila D. Fernández S. Torres-Alemán I. (2016). Astrocyte resilience to oxidative stress induced by insulin-like growth factor I (IGF-I) involves preserved AKT (protein kinase B) activity. J. Biol. Chem. 291, 12039. 10.1074/jbc.A115.695478 27261528 PMC4933256

[B61] de Barradas J. P. (1957). Plantas Mágicas Americanas. Consejo Superior de Investigaciones Científicas, Instituto “Bernardino de Sahagún.”

[B62] de Mejia E. G. Ramirez-Mares M. V. Puangpraphant S. (2009). Bioactive components of tea: cancer, inflammation and behavior. Brain. Behav. Immun. 23, 721–731. 10.1016/j.bbi.2009.02.013 19258034

[B63] Dhingra D. Parle M. Kulkarni S. K. (2004). Memory enhancing activity of Glycyrrhiza glabra in mice. J. Ethnopharmacol. 91, 361–365. 10.1016/j.jep.2004.01.016 15120462

[B64] Dkhar P. Sharma R. (2010). Effect of dimethylsulphoxide and curcumin on protein carbonyls and reactive oxygen species of cerebral hemispheres of mice as a function of age. Int. J. Dev. Neurosci. 28, 351–357. 10.1016/j.ijdevneu.2010.04.005 20403421

[B65] Doggui S. Belkacemi A. Paka G. D. Perrotte M. Pi R. Ramassamy C. (2013). Curcumin protects neuronal-like cells against acrolein by restoring Akt and redox signaling pathways. Mol. Nutr. Food Res. 57, 1660–1670. 10.1002/mnfr.201300130 23901044

[B66] Dolgin E. (2016). How to defeat dementia. Nature 539, 156–158. 10.1038/539156a 27830826

[B67] Domoráková I. Burda J. Mechírová E. Feriková M. (2006). Mapping of rat hippocampal neurons with NeuN after ischemia/reperfusion and *Ginkgo biloba* extract (EGb 761) pretreatment. Cell. Mol. Neurobiol. 26, 1191–1202. 10.1007/s10571-006-9080-6 16758319 PMC11520701

[B68] Dong S. Zeng Q. Mitchell E. S. Xiu J. Duan Y. Li C.. (2012). Curcumin enhances neurogenesis and cognition in aged rats: implications for transcriptional interactions related to growth and synaptic plasticity. PLoS ONE 7:e31211. 10.1371/journal.pone.0031211 22359574 PMC3281036

[B69] Doody R. S. Stevens J. C. Beck C. Dubinsky R. M. Kaye J. A. Gwyther L.. (2001). Practice parameter: management of dementia (an evidence-based review) Report of the Quality Standards Subcommittee of the American Academy of Neurology. Neurology 56, 1154–1166. 10.1212/WNL.56.9.1154 11342679

[B70] Dragicevic N. Smith A. Lin X. Yuan F. Copes N. Delic V.. (2011). Green tea epigallocatechin-3-gallate (EGCG) and other flavonoids reduce Alzheimer's amyloid-induced mitochondrial dysfunction. J. Alzheimers. Dis. 26, 507–521. 10.3233/JAD-2011-101629 21694462

[B71] Dringen R. (2000). Metabolism and functions of glutathione in brain. Prog. Neurobiol. 62, 649–671. 10.1016/S0301-0082(99)00060-X 10880854

[B72] Duke J. A. Ayensu E. S. (1985). Medicinal Plants of China. Algonac: Reference Publications.

[B73] Eckert A. Keil U. Scherping I. Hauptmann S. Muller W. E. (2005). Stabilization of mitochondrial membrane potential and improvement of neuronal energy metabolism by *Ginkgo biloba* extract EGb 761. Ann. N.Y. Acad. Sci. 1056, 474–485. 10.1196/annals.1352.023 16387710

[B74] Eckert G. P. Schiborr C. Hagl S. Abdel-Kader R. Muller W. E. Rimbach G.. (2013). Curcumin prevents mitochondrial dysfunction in the brain of the senescence-accelerated mouse-prone 8. Neurochem. Int. 62, 595–602. 10.1016/j.neuint.2013.02.014 23422877

[B75] Egashira N. Hayakawa K. Osajima M. Mishima K. Iwasaki K. Oishi R.. (2007). Involvement of GABA(A) receptors in the neuroprotective effect of theanine on focal cerebral ischemia in mice. J. Pharmacol. Sci. 105, 211–214. 10.1254/jphs.SCZ070901 17928735

[B76] Egashira N. Ishigami N. Pu F. Mishima K. Iwasaki K. Orito K.. (2008). Theanine prevents memory impairment induced by repeated cerebral ischemia in rats. Phytother. Res. 22, 65–68. 10.1002/ptr.2261 17705146

[B77] Ezoulin M. J. M. Ombetta J.-E. Dutertre-Catella H. Warnet J.-M. Massicot F. (2008). Antioxidative properties of galantamine on neuronal damage induced by hydrogen peroxide in SK-N-SH cells. Neurotoxicology 29, 270–277. 10.1016/j.neuro.2007.11.004 18191456

[B78] Farkas E. Luiten P. G. (2001). Cerebral microvascular pathology in aging and Alzheimer's disease. Prog. Neurobiol. 64, 575–611. 10.1016/S0301-0082(00)00068-X 11311463

[B79] Fatumbi P. V. P. (1995). Ewé: The Use of Plants in Yoruba Society. Salvador: Oderbrecht.

[B80] Favaloro B. Allocati N. Graziano V. Di Ilio C. De Laurenzi V. (2012). Role of Apoptosis in disease. Aging (Albany NY) 4, 330–349. 10.18632/aging.100459 22683550 PMC3384434

[B81] Finkler K. (1985). Spiritualist Healers in Mexico: Successes and Failures of Alternative Therapeutics. Salem, WI: Praeger Publishers.

[B82] Fox C. Crugel M. Maidment I. Auestad B. H. Coulton S. Treloar A.. (2012). Efficacy of memantine for agitation in Alzheimer's dementia: a randomised double-blind placebo controlled trial. PLoS ONE 7:e35185. 10.1371/journal.pone.0035185 22567095 PMC3342281

[B83] Franzblau M. Gonzales-Portillo C. Gonzales-Portillo G. S. Diamandis T. Borlongan M. C. Tajiri N.. (2013). Vascular damage: a persisting pathology common to Alzheimer's disease and traumatic brain injury. Med. Hypotheses 81, 842–845. 10.1016/j.mehy.2013.09.012 24074832 PMC3836590

[B84] Fuchs L. (1543). Kräuterbuch. Michael Isingrin. Basel.

[B85] Gao H.-M. Zhou H. Hong J.-S. (2014). Oxidative stress, neuroinflammation, and neurodegeneration, in Neuroinflammation, and Neurodegeneration, eds Peterson P. K. Toborek M. (New York, NY: Springer New York), 81–104.

[B86] Gate D. Rezai-Zadeh K. Jodry D. Rentsendorj A. Town T. (2010). Macrophages in Alzheimer's disease: the blood-borne identity. J. Neural Transm. 117, 961–970. 10.1007/s00702-010-0422-7 20517700 PMC2917548

[B87] Geeganage C. Wilcox R. Bath P. M. W. (2010). Triple antiplatelet therapy for preventing vascular events: a systematic review and meta-analysis. BMC Med. 8:36. 10.1186/1741-7015-8-36 20553581 PMC2893089

[B88] Giacobini E. (2004). Cholinesterase inhibitors: new roles and therapeutic alternatives. Pharmacol. Res. 50, 433–440. 10.1016/j.phrs.2003.11.017 15304240

[B89] Giri M. Zhang M. Lu Y. (2016). Genes associated with Alzheimer's disease: an overview and current status. Clin. Interv. Aging 11, 665–681. 10.2147/CIA.S105769 27274215 PMC4876682

[B90] Goenka P. Sarawgi A. Karun V. Nigam A. G. Dutta S. Marwah N. (2013). Camellia sinensis (Tea): implications and role in preventing dental decay. Pharmacogn. Rev. 7, 152–156. 10.4103/0973-7847.120515 24347923 PMC3841993

[B91] Gómez-Sámano M. Á. Grajales-Gómez M. Zuarth-Vázquez J. M. Navarro-Flores M. F. Martínez-Saavedra M. Juárez-León Ó. A.. (2017). Fibroblast growth factor 21 and its novel association with oxidative stress. Redox Biol. 11, 335–341. 10.1016/j.redox.2016.12.024 28039838 PMC5200873

[B92] González Ayala J. C. (1994). Botánica Medicinal Popular: Etnobotánica Medicinal de El Salvador. Jardín Botànico La Laguna.

[B93] Grand J. H. Caspar S. Macdonald S. W. (2011). Clinical features and multidisciplinary approaches to dementia care. J Multidiscip Heal. 2011, 125–147. 10.2147/JMDH.S17773 PMC3104685 21655340

[B94] Guan R. Zhao Y. Zhang H. Fan G. Liu X. Zhou W.. (2016). Draft genome of the living fossil *Ginkgo biloba*. Gigascience 5, 49. 10.1186/s13742-016-0154-1 27871309 PMC5118899

[B95] Gurib-Fakim A. (2006). Medicinal plants: traditions of yesterday and drugs of tomorrow. Mol. Aspects Med. 27, 1–93. 10.1016/j.mam.2005.07.008 16105678

[B96] Hajnoczky G. Davies E. Madesh M. (2003). Calcium signaling and apoptosis. Biochem. Biophys. Res. Commun. 304, 445–454. 10.1016/S0006-291X(03)00616-8 12729578

[B97] Hardy J. A. Higgins G. A. (1992). Alzheimer's disease: the amyloid cascade hypothesis. Science (256, 184. 1566067 10.1126/science.1566067

[B98] Haskell C. F. Kennedy D. O. Milne A. L. Wesnes K. A. Scholey A. B. (2008). The effects of L-theanine, caffeine and their combination on cognition and mood. Biol. Psychol. 77, 113–122. 10.1016/j.biopsycho.2007.09.008 18006208

[B99] Heim C. Khan M. A. Motsch B. Gocht A. Ramsperger-Gleixner M. Stamminger T. (2017). microvascular integrity can be preserved by anti-platelet therapy and in combination with mTOR inhibitor. J. Hear. Lung Transplant. 36, S376 10.1016/j.healun.2017.01.1069

[B100] Hikino H. (1985). Recent research on oriental medicinal plants. Econ. Med. Plant. Res. 1, 53–85.

[B101] Hoppe J. B. Coradini K. Frozza R. L. Oliveira C. M. Meneghetti A. B. Bernardi A.. (2013a). Free and nanoencapsulated curcumin suppress beta-amyloid-induced cognitive impairments in rats: involvement of BDNF and Akt/GSK-3beta signaling pathway. Neurobiol. Learn. Mem. 106, 134–144. 10.1016/j.nlm.2013.08.001 23954730

[B102] Hoppe J. B. Haag M. Whalley B. J. Salbego C. G. Cimarosti H. (2013b). Curcumin protects organotypic hippocampal slice cultures from Abeta1-42-induced synaptic toxicity. Toxicol. In Vitro 27, 2325–2330. 10.1016/j.tiv.2013.10.002 24134851

[B103] Howard R. J. Juszczak E. Ballard C. G. Bentham P. Brown R. G. Bullock R.. (2007). Donepezil for the treatment of agitation in Alzheimer's disease. N. Engl. J. Med. 357, 1382–1392. 10.1056/NEJMoa066583 17914039

[B104] Howes M.-J. R. Houghton P. J. (2003). Plants used in Chinese and Indian traditional medicine for improvement of memory and cognitive function. Pharmacol. Biochem. Behav. 75, 513–527. 10.1016/S0091-3057(03)00128-X 12895669

[B105] Howes M.-J. R. Perry E. (2011). The role of phytochemicals in the treatment and prevention of dementia. Drugs Aging 28, 439–468. 10.2165/11591310-000000000-00000 21639405

[B106] Howes M. R. Perry N. S. L. Houghton P. J. (2003). Plants with traditional uses and activities, relevant to the management of Alzheimer's disease and other cognitive disorders. Phyther. Res. 17, 1–18. 10.1002/ptr.1280 12557240

[B107] Huang H.-C. Tang D. Xu K. Jiang Z.-F. (2014). Curcumin attenuates amyloid-beta-induced tau hyperphosphorylation in human neuroblastoma SH-SY5Y cells involving PTEN/Akt/GSK-3beta signaling pathway. J. Recept. Signal Transduct. Res. 34, 26–37. 10.3109/10799893.2013.848891 24188406

[B108] Hügel H. M. Jackson N. (2015). Polyphenols for the prevention and treatment of dementia diseases. Neural Regen. Res. 10, 1756–1758. 10.4103/1673-5374.169609 26807106 PMC4705783

[B109] Huminiecki L. Horbanczuk J. Atanasov A. G. (2017). The functional genomic studies of curcumin. Semin. Cancer Biol. 46, 107–118. 10.1016/j.semcancer.2017.04.002 28392463

[B110] Hung C. H.-L. Cheng S. S.-Y. Cheung Y.-T. Wuwongse S. Zhang N. Q. Ho Y.-S.. (2018). A reciprocal relationship between reactive oxygen species and mitochondrial dynamics in neurodegeneration. Redox Biol. 14, 7–19. 10.1016/j.redox.2017.08.010 28837882 PMC5567977

[B111] Hurd M. D. Martorell P. Langa K. M. (2013). Monetary costs of dementia in the United States. N. Engl. J. Med. 369, 489–490. 10.1056/NEJMc1305541 23902508

[B112] Iadecola C. (2013). The pathobiology of vascular dementia. Neuron 80, 844–866. 10.1016/j.neuron.2013.10.008 24267647 PMC3842016

[B113] IARC Working Group (2016). Some Drugs and Herbal Products. Lyon: Ginkgo Biloba. International Agency for Research and Cancer (IARC) Monographs, WHO.

[B114] Ibrahim F. Knight S. R. Cramer R. L. (2012). Addressing the controversial use of antipsychotic drugs for behavioral and psychological symptoms of dementia. J. Pharm. Technol. 28, 3–9. 10.1177/875512251202800102

[B115] Ihl R. Tribanek M. Bachinskaya N. (2012). Efficacy and tolerability of a once daily formulation of *Ginkgo biloba* extract EGb 761(R) in Alzheimer's disease and vascular dementia: results from a randomised controlled trial. Pharmacopsychiatry 45, 41–46. 10.1055/s-0031-1291217 22086747

[B116] Ikarashi Y. Mizoguchi K. (2016). Neuropharmacological efficacy of the traditional Japanese Kampo medicine yokukansan and its active ingredients. Pharmacol. Ther. 166, 84–95. 10.1016/j.pharmthera.2016.06.018 27373856

[B117] Iqbal K. Liu F. Gong C.-X. (2016). Tau and neurodegenerative disease: the story so far. Nat. Rev. Neurol. 12, 15–27. 10.1038/nrneurol.2015.225 26635213

[B118] Iqbal K. Liu F. Gong C.-X. Grundke-Iqbal I. (2010). Tau in Alzheimer disease and related tauopathies. Curr. Alzheimer Res. 7, 656–664. 10.2174/156720510793611592 20678074 PMC3090074

[B119] Isah T. (2015). Rethinking *Ginkgo biloba* L.: medicinal uses and conservation. Pharmacogn. Rev. 9, 140–148. 10.4103/0973-7847.162137 26392712 PMC4557237

[B120] Izzo A. A. (2012). Interactions between herbs and conventional drugs: overview of the clinical data. Med. Princ. Pract. 21, 404–428. 10.1159/000334488 22236736

[B121] Jahanshahi M. Nickmahzar E. G. Babakordi F. (2013). Effect of Gingko biloba extract on scopolamine-induced apoptosis in the hippocampus of rats. Anat. Sci. Int. 88, 217–222. 10.1007/s12565-013-0188-8 23828103

[B122] Jahanshahi M. Nikmahzar E. Yadollahi N. Ramazani K. (2012). Protective effects of *Ginkgo biloba* extract (EGB 761) on astrocytes of rat hippocampus after exposure with scopolamine. Anat. Cell Biol. 45, 92–96. 10.5115/acb.2012.45.2.92 22822463 PMC3398180

[B123] Jelenkovic A. Jovanovic M. D. Stevanovic I. Petronijevic N. Bokonjic D. Zivkovic J.. (2014). Influence of the green tea leaf extract on neurotoxicity of aluminium chloride in rats. Phytother. Res. 28, 82–87. 10.1002/ptr.4962 23494944

[B124] Ju H. S. Li X. J. Zhao B. L. Han Z. W. Xin W. J. (1989). Effects of glycyrrhiza flavonoid on lipid peroxidation and active oxygen radicals. Yao Xue Xue Bao 24, 807–812. 2618676

[B125] Kanno H. Kawakami Z. Tabuchi M. Mizoguchi K. Ikarashi Y. Kase Y. (2015). Protective effects of glycycoumarin and procyanidin B1, active components of traditional Japanese medicine yokukansan, on amyloid beta oligomer-induced neuronal death. J. Ethnopharmacol. 159, 122–128. 10.1016/j.jep.2014.10.058 25446602

[B126] Kanowski S. Herrmann W. M. Stephan K. Wierich W. Horr R. (1996). Proof of efficacy of the *Ginkgo biloba* special extract EGb 761 in outpatients suffering from mild to moderate primary degenerative dementia of the Alzheimer type or multi-infarct dementia. Pharmacopsychiatry 29, 47–56. 10.1055/s-2007-979544 8741021

[B127] Katsel P. Tan W. Fam P. Purohit D. P. Haroutunian V. (2013). Cell cycle checkpoint abnormalities during dementia: a plausible association with the loss of protection against oxidative stress in Alzheimer's disease [corrected]. PLoS ONE 8:e68361. 10.1371/journal.pone.0068361 23861893 PMC3702571

[B128] Kaur U. Banerjee P. Bir A. Sinha M. Biswas A. Chakrabarti S. (2015). Reactive oxygen species, redox signaling and neuroinflammation in Alzheimer's disease: the NF-kappaB connection. Curr. Top. Med. Chem. 15, 446–457. 10.2174/1568026615666150114160543 25620241

[B129] Kihara T. Shimohama S. (2004). Alzheimer's disease and acetylcholine receptors. Acta Neurobiol. Exp. (Wars). 64, 99–105. 15190684 10.55782/ane-2004-1495

[B130] Kim J. M. Ryou S. H. Kang Y. H. Kang J. S. (2011). Effect of *Ginkgo biloba* leaf powder and extract on plasma and liver lipids, platelet aggregation and erythrocyte Na+ efflux in rats fed hypercholesterolemic diet. FASEB J. 25, 980–988.

[B131] Kim J. Shim J. Lee S. Cho W.-H. Hong E. Lee J. H.. (2016). Rg3-enriched ginseng extract ameliorates scopolamine-induced learning deficits in mice. BMC Complement. Altern. Med. 16:66. 10.1186/s12906-016-1050-z 26887326 PMC4758096

[B132] Kim M.-S. Lee J.-I. Lee W.-Y. Kim S.-E. (2004). Neuroprotective effect of *Ginkgo biloba* L. extract in a rat model of Parkinson's disease. Phytother. Res. 18, 663–666. 10.1002/ptr.1486 15472919

[B133] Kim T. I. Lee Y. K. Park S. G. Choi I. S. Ban J. O. Park H. K.. (2009). l-Theanine, an amino acid in green tea, attenuates beta-amyloid-induced cognitive dysfunction and neurotoxicity: reduction in oxidative damage and inactivation of ERK/p38 kinase and NF-kappaB pathways. Free Radic. Biol. Med. 47, 1601–1610. 10.1016/j.freeradbiomed.2009.09.008 19766184

[B134] Kim Y. C. Kim S. R. Markelonis G. J. Oh T. H. (1998). Ginsenosides Rb1 and Rg3 protect cultured rat cortical cells from glutamate-induced neurodegeneration. J. Neurosci. Res. 53, 426–432. 9710262 10.1002/(SICI)1097-4547(19980815)53:4<426::AID-JNR4>3.0.CO;2-8

[B135] Kimura K. Ozeki M. Juneja L. R. Ohira H. (2007). L-Theanine reduces psychological and physiological stress responses. Biol. Psychol. 74, 39–45. 10.1016/j.biopsycho.2006.06.006 16930802

[B136] Knapp M. Prince M. Albanese E. Banerjee S. Dhanasiri S. Fernandez J. L. (2007). Dementia UK: The Full Report. London: Alzheimer's Society.

[B137] Koola M. M. Buchanan R. W. Pillai A. Aitchison K. J. Weinberger D. R. Aaronson S. T.. (2014). Potential role of the combination of galantamine and memantine to improve cognition in schizophrenia. Schizophr. Res. 157, 84–89. 10.1016/j.schres.2014.04.037 24878431 PMC4099270

[B138] Kumar A. Prakash A. Pahwa D. (2011). Galantamine potentiates the protective effect of rofecoxib and caffeic acid against intrahippocampal Kainic acid-induced cognitive dysfunction in rat. Brain Res. Bull. 85, 158–168. 10.1016/j.brainresbull.2011.03.010 21439356

[B139] Kumar A. Singh A. (2015). A review on mitochondrial restorative mechanism of antioxidants in Alzheimer's disease and other neurological conditions. Front. Pharmacol. 6:206. 10.3389/fphar.2015.00206 26441662 PMC4585235

[B140] LeBlanc A. C. (2005). The role of apoptotic pathways in Alzheimer's disease neurodegeneration and cell death. Curr. Alzheimer Res. 2, 389–402. 10.2174/156720505774330573 16248844

[B141] Lee J. Kim Y. Liu T. Hwang Y. J. Hyeon S. J. Im H.. (2017). SIRT3 deregulation is linked to mitochondrial dysfunction in Alzheimer's disease. Aging Cell. [Epub ahead of print]. 10.1111/acel.12679 29130578 PMC5771400

[B142] Lee T.-K. Johnke R. M. Allison R. R. O'Brien K. F. Dobbs L. J. J. (2005). Radioprotective potential of ginseng. Mutagenesis 20, 237–243. 10.1093/mutage/gei041 15956041

[B143] Li W. Chu Y. Zhang L. Yin L. Li L. (2012). Ginsenoside Rg1 attenuates tau phosphorylation in SK-N-SH induced by Abeta-stimulated THP-1 supernatant and the involvement of p38 pathway activation. Life Sci. 91, 809–815. 10.1016/j.lfs.2012.08.028 22982182

[B144] Lilienfeld S. (2002). Galantamine–a novel cholinergic drug with a unique dual mode of action for the treatment of patients with Alzheimer's disease. CNS Drug Rev. 8, 159–176. 10.1111/j.1527-3458.2002.tb00221.x 12177686 PMC6741688

[B145] Lim S. L. Rodriguez-Ortiz C. J. Kitazawa M. (2015). Infection, systemic inflammation, and Alzheimer's disease. Microbes Infect. 17, 549–556. 10.1016/j.micinf.2015.04.004 25912134

[B146] Lin M. T. Beal M. F. (2006). Mitochondrial dysfunction and oxidative stress in neurodegenerative diseases. Nature 443, 787–795. 10.1038/nature05292 17051205

[B147] Liu C.-C. Liu C.-C. Kanekiyo T. Xu H. Bu G. (2013). Apolipoprotein E and Alzheimer disease: risk, mechanisms and therapy. Nat. Rev. Neurol. 9, 106–118. 10.1038/nrneurol.2012.263 23296339 PMC3726719

[B148] Liu X. Xu K. Yan M. Wang Y. Zheng X. (2010). Protective effects of galantamine against Aβ-induced PC12 cell apoptosis by preventing mitochondrial dysfunction and endoplasmic reticulum stress. Neurochem. Int. 57, 588–599. 10.1016/j.neuint.2010.07.007 20655346

[B149] Long F. Yang H. Xu Y. Hao H. Li P. (2015). A strategy for the identification of combinatorial bioactive compounds contributing to the holistic effect of herbal medicines. Sci. Rep. 5:12361. 10.1038/srep12361 26198093 PMC4510521

[B150] Lonicerus A. (1679). Kräuterbuch, Matthias Wagner. Ulm.

[B151] Loy C. Schneider L. (2006). Galantamine for Alzheimer's disease and mild cognitive impairment. Cochrane database Syst. Rev. 25:CD001747 10.1002/14651858.CD001747.pub3 PMC8961200 16437436

[B152] Luengo-Fernandez R. Leal J. Gray A. (2010). Dementia 2010: The Economic Burden of Dementia and Associated Research Funding in the United Kingdom. Oxford: Cambridge Alzheimer's Research Trust.

[B153] Ma B. Meng X. Wang J. Sun J. Ren X. Qin M.. (2014). Notoginsenoside R1 attenuates amyloid-beta-induced damage in neurons by inhibiting reactive oxygen species and modulating MAPK activation. Int. Immunopharmacol. 22, 151–159. 10.1016/j.intimp.2014.06.018 24975829

[B154] Mahley R. W. Rall S. C. Jr. (2000). Apolipoprotein E: far more than a lipid transport protein. Annu. Rev. Genomics Hum. Genet. 1, 507–537. 10.1146/annurev.genom.1.1.507 11701639

[B155] Mahmood T. Akhtar N. Khan B. A. (2010). The morphology, characteristics, and medicinal properties of Camellia sinensis tea. J. Med. Plants Res. 4, 2028–2033. 10.5897/JMPR10.010

[B156] Mandel S. A. Amit T. Weinreb O. Youdim M. B. H. (2011). Understanding the broad-spectrum neuroprotective action profile of green tea polyphenols in aging and neurodegenerative diseases. J. Alzheimers. Dis. 25, 187–208. 10.3233/JAD-2011-101803 21368374

[B157] Mantle D. Pickering A. T. Perry E. K. (2000). Medicinal plant extracts for the treatment of dementia. CNS Drugs 13, 201–213. 10.2165/00023210-200013030-00006

[B158] Manyam B. V. (1999). Dementia in ayurveda. J. Altern. Complement. Med. 5, 81–88. 10.1089/acm.1999.5.81 10100034

[B159] McKhann G. M. Albert M. S. Grossman M. Miller B. Dickson D. Trojanowski J. Q. (2001). Clinical and pathological diagnosis of frontotemporal dementia: report of the Work Group on Frontotemporal Dementia and Pick's Disease. Arch. Neurol. 58, 1803–1809. 10.1001/archneur.58.11.1803 11708987

[B160] Mehan S. Sharma D. Sharma G. Arora R. Sehgal V. (2012). Dementia-A Complete Literature Review on Various Mechanisms Involves in Pathogenesis and an Intracerebroventricular Streptozotocin Induced Alzheimer's Disease. INTECH Open Access Publisher Available online at: http://www.intechopen.com/books/inflammatory-diseases-immunopathology-clinical-and-pharmacologicalbases/alzheimer-s-disease-an-updated-review-on-pathogenesis-and-intracerebroventricular-streptozotocin-ind

[B161] Miao Y. Zhao S. Gao Y. Wang R. Wu Q. Wu H.. (2016). Curcumin pretreatment attenuates inflammation and mitochondrial dysfunction in experimental stroke: the possible role of Sirt1 signaling. Brain Res. Bull. 121, 9–15. 10.1016/j.brainresbull.2015.11.019 26639783

[B162] Mills S. Y. (1991). Out of the Earth: The Essential Book of Herbal Medicine. London: Viking.

[B163] Mishra S. Palanivelu K. (2008). The effect of curcumin (turmeric) on Alzheimer's disease: an overview. Ann. Indian Acad. Neurol. 11, 13–19. 10.4103/0972-2327.40220 19966973 PMC2781139

[B164] Misra R. (1998). Modern drug development from traditional medicinal plants using radioligand receptor-binding assays. Med. Res. Rev. 18, 383–402. 9828039 10.1002/(sici)1098-1128(199811)18:6<383::aid-med3>3.0.co;2-a

[B165] Monsch A. U. Giannakopoulos P. (2004). Effects of galantamine on behavioural and psychological disturbances and caregiver burden in patients with Alzheimer's disease. Curr. Med. Res. Opin. 20, 931–938. 10.1185/030079904125003890 15200752

[B166] Moreira P. I. Carvalho C. Zhu X. Smith M. A. Perry G. (2010). Mitochondrial dysfunction is a trigger of Alzheimer's disease pathophysiology. Biochim. Biophys. Acta 1802, 2–10. 10.1016/j.bbadis.2009.10.006 19853658

[B167] Müller-Ebeling C. Rätsch C. (1989). Heilpflanzen der Seychellen.VWB-Verlag für Wissenschaft und Bildung. Berlin.

[B168] Nah S.-Y. (2014). Ginseng ginsenoside pharmacology in the nervous system: involvement in the regulation of ion channels and receptors. Front. Physiol. 5:98. 10.3389/fphys.2014.00098 24678300 PMC3958645

[B169] Namanja H. A. Emmert D. Pires M. M. Hrycyna C. A. Chmielewski J. (2009). Inhibition of human P-glycoprotein transport and substrate binding using a galantamine dimer. Biochem. Biophys. Res. Commun. 388, 672–676. 10.1016/j.bbrc.2009.08.056 19683513 PMC2764298

[B170] Napryeyenko O. Sonnik G. Tartakovsky I. (2009). Efficacy and tolerability of *Ginkgo biloba* extract EGb 761 by type of dementia: analyses of a randomised controlled trial. J. Neurol. Sci. 283, 224–229. 10.1016/j.jns.2009.02.353 19286192

[B171] Narlawar R. Pickhardt M. Leuchtenberger S. Baumann K. Krause S. Dyrks T.. (2008). Curcumin-derived pyrazoles and isoxazoles: swiss army knives or blunt tools for Alzheimer's disease? ChemMedChem 3, 165–172. 10.1002/cmdc.200700218 17943713

[B172] Navia B. A. Rostasy K. (2005). The AIDS dementia complex: clinical and basic neuroscience with implications for novel molecular therapies. Neurotox. Res. 8, 3–24. 10.1007/BF03033817 16260383

[B173] Ng T.-P. Chiam P.-C. Lee T. Chua H.-C. Lim L. Kua E.-H. (2006). Curry consumption and cognitive function in the elderly. Am. J. Epidemiol. 164, 898–906. 10.1093/aje/kwj267 16870699

[B174] Ng Y. P. Or T. C. T. Ip N. Y. (2015). Plant alkaloids as drug leads for Alzheimer's disease. Neurochem. Int. 89, 260–270. 10.1016/j.neuint.2015.07.018 26220901

[B175] Ngan F. Shaw P. But P. Wang J. (1999). Molecular authentication of Panax species. Phytochemistry 50, 787–791. 10.1016/S0031-9422(98)00606-2 10192964

[B176] Niikura T. Tajima H. Kita Y. (2006). Neuronal cell death in Alzheimer's disease and a neuroprotective factor, humanin. Curr. Neuropharmacol. 4, 139–147. 10.2174/157015906776359577 18615127 PMC2430668

[B177] Nimmrich V. Eckert A. (2013). Calcium channel blockers and dementia. Br. J. Pharmacol. 169, 1203–1210. 10.1111/bph.12240 23638877 PMC3831702

[B178] Nishida K. Yasuda E. Nagasawa K. Fujimoto S. (2008). Altered levels of oxidation and phospholipase C isozyme expression in the brains of theanine-administered rats. Biol. Pharm. Bull. 31, 857–860. 10.1248/bpb.31.857 18451507

[B179] Nishiyama N. Chu P. J. Saito H. (1995). Beneficial effects of Biota, a traditional Chinese herbal medicine on learning impairment induced by basal forebrain-lesion in mice. Biol. Pharm. Bull. 28, 1513–1517. 10.1248/bpb.18.1513 8593469

[B180] Olin J. Schneider L. (2002). Galantamine for Alzheimer's disease. Cochrane database Syst. Rev. 3:CD001747 10.1002/14651858.CD001747 12137632

[B181] Ortiz de Montellano B. R. (1990). Aztec Medicine, Health and Nutrition. New Brunswick: Rutgers University Press.

[B182] Owen G. N. Parnell H. De Bruin E. A. Rycroft J. A. (2008). The combined effects of L-theanine and caffeine on cognitive performance and mood. Nutr. Neurosci. 11, 193–198. 10.1179/147683008X301513 18681988

[B183] Paganelli R. A. Benetoli A. Milani H. (2006). Sustained neuroprotection and facilitation of behavioral recovery by the *Ginkgo biloba* extract, EGb 761, after transient forebrain ischemia in rats. Behav. Brain Res. 174, 70–77. 10.1016/j.bbr.2006.07.005 16934342

[B184] Parle M. Dhingra D. Kulkarni S. K. (2004). Memory-strengthening activity of Glycyrrhiza glabra in exteroceptive and interoceptive behavioral models. J. Med. Food 7, 462–466. 10.1089/jmf.2004.7.462 15671690

[B185] Pasqualetti G. Brooks D. J. Edison P. (2015). The role of neuroinflammation in dementias. Curr. Neurol. Neurosci. Rep. 15:17. 10.1007/s11910-015-0531-7 25716012

[B186] Pastor P. Roe C. M. Villegas A. Bedoya G. Chakraverty S. Garcia G.. (2003). Apolipoprotein Eepsilon4 modifies Alzheimer's disease onset in an E280A PS1 kindred. Ann. Neurol. 54, 163–169. 10.1002/ana.10636 12891668

[B187] Pathak L. Agrawal Y. Dhir A. (2013). Natural polyphenols in the management of major depression. Expert Opin. Investig. Drugs 22, 863–880. 10.1517/13543784.2013.794783 23642183

[B188] Perry E. Howes M. R. (2011). Medicinal plants and dementia therapy: herbal hopes for brain aging? CNS Neurosci. Ther. 17, 683–698. 10.1111/j.1755-5949.2010.00202.x 22070157 PMC6493900

[B189] Perry E. K. Pickering A. T. Wang W. W. Houghton P. Perry N. S. L. (1998). Medicinal plants and Alzheimer's disease: integrating ethnobotanical and contemporary scientific evidence. J. Altern. Complement. Med. 4, 419–428. 9884179 10.1089/acm.1998.4.419

[B190] Pohl S. Zobel J. Moffat A. (2010). Extended boolean retrieval for systematic biomedical reviews, in Proceedings of the Thirty-Third Australasian Conferenc on Computer Science - Volume 102 ACSC'10. (Darlinghurst, NSW: Australian Computer Society, Inc), 117–126. Available online at: http://dl.acm.org/citation.cfm?id=1862199.1862212

[B191] Popovich D. G. Kitts D. D. (2004). Generation of ginsenosides Rg3 and Rh2 from North American ginseng. Phytochemistry 65, 337–344. 10.1016/j.phytochem.2003.11.020 14751305

[B192] Power M. C. Adar S. D. Yanosky J. D. Weuve J. (2016). Exposure to air pollution as a potential contributor to cognitive function, cognitive decline, brain imaging, and dementia: a systematic review of epidemiologic research. Neurotoxicology 56, 235–253. 10.1016/j.neuro.2016.06.004 27328897 PMC5048530

[B193] Price S. Price L. (1995). Aromatherapy for Health Professionals. Edinburgh: Churchill Livingston.

[B194] Prince M. Comas-Herrera A. Knapp M. Guerchet M. Karagiannidou M. (2016). World Alzheimer Report 2016: Improving Healthcare for People Living with Dementia: Coverage, Quality and Costs Now and in the Future. London: Personal Social Services Research Unit; London School of Economics and Political Science.

[B195] Qiao X. Ji S. Yu S.-W. Lin X.-H. Jin H.-W. Duan Y.-K.. (2014). Identification of key licorice constituents which interact with cytochrome P450: evaluation by LC/MS/MS cocktail assay and metabolic profiling. AAPS J. 16, 101–113. 10.1208/s12248-013-9544-9 24254844 PMC3889530

[B196] Qiu C. Kivipelto M. von Strauss E. (2009). Epidemiology of Alzheimer's disease: occurrence, determinants, and strategies toward intervention. Dialogues Clin. Neurosci. 11, 111–128. 19585947 10.31887/DCNS.2009.11.2/cqiu PMC3181909

[B197] Quaglio G. Brand H. Dario C. (2016). Fighting dementia in Europe: the time to act is now. Lancet Neurol. 15, 452–454. 10.1016/S1474-4422(16)00079-X 26987700

[B198] Rabinovici G. D. Miller B. L. (2010). Frontotemporal lobar degeneration: epidemiology, pathophysiology, diagnosis and management. CNS Drugs 24, 375–398. 10.2165/11533100-000000000-00000 20369906 PMC2916644

[B199] Raina A. K. Zhu X. Rottkamp C. A. Monteiro M. Takeda A. Smith M. A. (2000). Cyclin' toward dementia: cell cycle abnormalities and abortive oncogenesis in Alzheimer disease. J. Neurosci. Res. 61, 128–133. 10.1002/1097-4547(20000715)61:2<128::AID-JNR2>3.0.CO;2-H 10878584

[B200] Ramkumar M. Rajasankar S. Gobi V. V. Dhanalakshmi C. Manivasagam T. Justin Thenmozhi A.. (2017). Neuroprotective effect of Demethoxycurcumin, a natural derivative of Curcumin on rotenone induced neurotoxicity in SH-SY 5Y Neuroblastoma cells. BMC Complement. Altern. Med. 17, 217. 10.1186/s12906-017-1720-5 28420370 PMC5395846

[B201] Rastogi M. Ojha R. P. Sagar C. Agrawal A. Dubey G. P. (2014). Protective effect of curcuminoids on age-related mitochondrial impairment in female Wistar rat brain. Biogerontology 15, 21–31. 10.1007/s10522-013-9466-z 24048922

[B202] Reddy A. P. Reddy P. H. (2017). mitochondria-targeted molecules as potential drugs to treat patients with Alzheimer's disease. Prog. Mol. Biol. Transl. Sci. 146, 173–201. 10.1016/bs.pmbts.2016.12.010 28253985

[B203] Rezai-Zadeh K. Arendash G. W. Hou H. Fernandez F. Jensen M. Runfeldt M.. (2008). Green tea epigallocatechin-3-gallate (EGCG) reduces beta-amyloid mediated cognitive impairment and modulates tau pathology in Alzheimer transgenic mice. Brain Res. 1214, 177–187. 10.1016/j.brainres.2008.02.107 18457818

[B204] Rhein V. Giese M. Baysang G. Meier F. Rao S. Schulz K. L.. (2010). *Ginkgo biloba* extract ameliorates oxidative phosphorylation performance and rescues abeta-induced failure. PLoS ONE 5:e12359. 10.1371/journal.pone.0012359 20808761 PMC2927422

[B205] Ribeiro M. L. Moreira L. M. Arcari D. P. Dos Santos L. F. Marques A. C. Pedrazzoli J. J.. (2016). Protective effects of chronic treatment with a standardized extract of *Ginkgo biloba* L. in the prefrontal cortex and dorsal hippocampus of middle-aged rats. Behav. Brain Res. 313, 144–150. 10.1016/j.bbr.2016.06.029 27424157

[B206] Ridley N. J. Draper B. Withall A. (2013). Alcohol-related dementia: an update of the evidence. Alzheimers. Res. Ther. 5, 3. 10.1186/alzrt157 23347747 PMC3580328

[B207] Rivas-Arancibia S. Hernández-Zimbrón L. F. Rodríguez-Martínez E. Borgonio-Pérez G. Velumani V. Durán-Bedolla J. (2013). Chronic exposure to low doses of ozone produces a state of oxidative stress and blood-brain barrier damage in the hippocampus of rat. Adv. Biosci. Biotechnol. 4:24 10.4236/abb.2013.411A2004

[B208] Roeder B.A. (1988). Chicano Folk Medicine from Los Angeles. Berkeley; Los Angeles: CA: University of California Press.

[B209] Romero A. Egea J. Garcia A. G. Lopez M. G. (2010). Synergistic neuroprotective effect of combined low concentrations of galantamine and melatonin against oxidative stress in SH-SY5Y neuroblastoma cells. J. Pineal Res. 49, 141–148. 10.1111/j.1600-079X.2010.00778.x 20536682

[B210] Ross I. A. (2001). Medicinal Plants of the World: Chemical Constituents, Traditional and Modern Medicinal Uses. New York, NY: Humana Press.

[B211] Ruttkay-Nedecky B. Nejdl L. Gumulec J. Zitka O. Masarik M. Eckschlager T.. (2013). The role of metallothionein in oxidative stress. Int. J. Mol. Sci. 14, 6044–6066. 10.3390/ijms14036044 23502468 PMC3634463

[B212] Saharan S. Mandal P. K. (2014). The emerging role of glutathione in Alzheimer's disease. J. Alzheimers. Dis. 40, 519–529. 10.3233/JAD-132483 24496077

[B213] Sahne F. Mohammadi M. Najafpour G. D. Moghadamnia A. A. (2017). Enzyme-assisted ionic liquid extraction of bioactive compound from turmeric (*Curcuma longa* L.): isolation, purification and analysis of curcumin. Ind. Crops Prod. 95, 686–694. 10.1016/j.indcrop.2016.11.037

[B214] Sama D. M. Norris C. M. (2013). Calcium dysregulation and neuroinflammation: discrete and integrated mechanisms for age-related synaptic dysfunction. Ageing Res. Rev. 12, 982–995. 10.1016/j.arr.2013.05.008 23751484 PMC3834216

[B215] Savelev S. U. Okello E. J. Perry E. K. (2004). Butyryl-and acetyl-cholinesterase inhibitory activities in essential oils of Salvia species and their constituents. Phyther. Res. 18, 315–324. 10.1002/ptr.1451 15162368

[B216] Schultes R. E. (1993). Plants in treating senile dementia in the Northwest Amazon. J. Ethnopharmacol. 38, 121–128. 10.1016/0378-8741(93)90007-R 8510460

[B217] Schultes R. E. (1994). Amazonian Ethnobotany and the Search for New Drugs BT - Ciba Foundation Symposium 185. Chichester: Wiley. 10.1002/9780470514634.ch8 7736849

[B218] Schulz R. McGinnis K. A. Zhang S. Martire L. M. Hebert R. S. Beach S. R.. (2008). Dementia patient suffering and caregiver depression. Alzheimer Dis. Assoc. Disord. 22, 170–176. 10.1097/WAD.0b013e31816653cc 18525290 PMC2782456

[B219] Schwarzkopf T. M. Koch K. A. Klein J. (2013). Neurodegeneration after transient brain ischemia in aged mice: beneficial effects of bilobalide. Brain Res. 1529, 178–187. 10.1016/j.brainres.2013.07.003 23850645

[B220] Schweitzer de Palacios D. (1994). Cambiashun. Las prácticas médicas tradicionales y sus expertos en San Miguel del Común, una comuna indí*gena en los alrededores de Quito* Berlin: Holos-Verlag; Free University of Berlin.

[B221] Sfikas G. (1980). Heilpflanzen Griechenlands. P. Efstathiadis & Söhne AG. Athen.

[B222] Shankar G. M. Walsh D. M. (2009). Alzheimer's disease: synaptic dysfunction and Aβ. Mol. Neurodegener. 4:48. 10.1186/1750-1326-4-48 19930651 PMC2788538

[B223] Sirirugsa P. Larsen K. Maknoi C. (2007). The genus *Curcuma* L.(zingiberaceae): distribution and classification with reference to species diversity in Thailand. Gard. Bull. Singapore 59, 203–320.

[B224] Solfrizzi V. Panza F. (2015). Plant-based nutraceutical interventions against cognitive impairment and dementia: meta-analytic evidence of efficacy of a standardized Gingko biloba extract. J. Alzheimers. Dis. 43, 605–611. 10.3233/JAD-141887 25352453

[B225] Song L. Xu M.-B. Zhou X.-L. Zhang D.-P. Zhang S.-L. Zheng G.-Q. (2017). A Preclinical systematic review of ginsenoside-Rg1 in experimental Parkinson's disease. Oxid. Med. Cell. Longev. 2017:2163053. 10.1155/2017/2163053 28386306 PMC5366755

[B226] Spano M. Signorelli M. Vitaliani R. Aguglia E. Giometto B. (2015). The possible involvement of mitochondrial dysfunctions in Lewy body dementia: a systematic review. Funct. Neurol. 30, 151–158. 10.11138/FNeur/2015.30.3.151 26346695 PMC4610749

[B227] Stackman R. W. Eckenstein F. Frei B. Kulhanek D. Nowlin J. Quinn J. F. (2003). Prevention of age-related spatial memory deficits in a transgenic mouse model of Alzheimer's disease by chronic *Ginkgo biloba* treatment. Exp. Neurol. 184, 510–520. 10.1016/S0014-4886(03)00399-6 14637120

[B228] Stafford G. I. Pedersen M. E. van Staden J. Jäger A. K. (2008). Review on plants with CNS-effects used in traditional South African medicine against mental diseases. J. Ethnopharmacol. 119, 513–537. 10.1016/j.jep.2008.08.010 18775771

[B229] Sun M. Ye Y. Xiao L. Duan X. Zhang Y. Zhang H. (2017). Anticancer effects of ginsenoside Rg3 (Review). Int. J. Mol. Med. 39, 507–518. 10.3892/ijmm.2017.2857 28098857

[B230] Sun S. Wang C.-Z. Tong R. Li X.-L. Fishbein A. Wang Q. (2010). Effects of steaming the root of Panax notoginseng on chemical composition and anticancer activities. Food Chem. 118, 307–314. 10.1016/j.foodchem.2009.04.122

[B231] Sun Z.-K. Yang H.-Q. Chen S.-D. (2013). Traditional Chinese medicine: a promising candidate for the treatment of Alzheimer's disease. Transl. Neurodegener. 2:6. 10.1186/2047-9158-2-6 23445907 PMC3599149

[B232] Swerdlow R. H. Burns J. M. Khan S. M. (2010). The Alzheimer's disease mitochondrial cascade hypothesis. J. Alzheimers. Dis. 20(Suppl. 2), S265–S279. 10.3233/JAD-2010-100339 20442494 PMC2883665

[B233] Swerdlow R. H. Koppel S. Weidling I. Hayley C. Ji Y. Wilkins H. M. (2017). mitochondria, cybrids, aging, and Alzheimer's disease. Prog. Mol. Biol. Transl. Sci. 146, 259–302. 10.1016/bs.pmbts.2016.12.017 28253988 PMC5864124

[B234] Tabernaemontanus D. I. T. (1987). Kräuterbuch, Johann Ludwig König/Johann Brandmüller. Basel.

[B235] Takuma K. Hoshina Y. Arai S. Himeno Y. Matsuo A. Funatsu Y.. (2007). *Ginkgo biloba* extract EGb 761 attenuates hippocampal neuronal loss and cognitive dysfunction resulting from chronic restraint stress in ovariectomized rats. Neuroscience 149, 256–262. 10.1016/j.neuroscience.2007.07.042 17869007

[B236] Tanaka A. Arai Y. Kim S.-N. Ham J. Usuki T. (2011). Synthesis and biological evaluation of bilobol and adipostatin A. J. Asian Nat. Prod. Res. 13, 290–296. 10.1080/10286020.2011.554828 21462031

[B237] Taylor L. (1998). Herbal Secrets of the Rainforest: The Healing Power of over 50 Medicinal Plants You Should Know About. Rocklin, CA: Prima Publishing.

[B238] Tayyem R. F. Heath D. D. Al-Delaimy W. K. Rock C. L. (2006). Curcumin content of turmeric and curry powders. Nutr. Cancer 55, 126–131. 10.1207/s15327914nc5502_2 17044766

[B239] Tewari D. Mocan A. Parvanov E. D. Sah A. N. Nabavi S. M. Huminiecki L. (2017). Ethnopharmacological approaches for therapy of jaundice: Part II. Highly used plant species from Acanthaceae, Euphorbiaceae, Asteraceae, Combretaceae, and Fabaceae families. Front. Pharmacol. 8:519 10.3389/fphar.2017.00518 28848436 PMC5554347

[B240] Tian J. Zhang S. Li G. Liu Z. Xu B. (2009). 20(S)-ginsenoside Rg3, a neuroprotective agent, inhibits mitochondrial permeability transition pores in rat brain. Phytother. Res. 23, 486–491. 10.1002/ptr.2653 19003949

[B241] Tripathi M. Vibha D. (2009). Reversible dementias. Indian J. Psychiatry 51, S52. 21416018 PMC3038529

[B242] Tsvetkova D. Obreshkova D. Zheleva-Dimitrova D. Saso L. (2013). Antioxidant activity of galantamine and some of its derivatives. Curr. Med. Chem. 20, 4595–4608. 10.2174/09298673113209990148 23834167

[B243] Tzvetkov N. T. Antonov L. (2017). Subnanomolar indazole-5-carboxamide inhibitors of monoamine oxidase B (MAO-B) continued: indications of iron binding, experimental evidence for optimised solubility and brain penetration. J. Enzyme Inhib. Med. Chem. 32, 960–967. 10.1080/14756366.2017.1344980 28726524 PMC6445166

[B244] van Horssen J. van Schaik P. Witte M. (2017). Inflammation and mitochondrial dysfunction: a vicious circle in neurodegenerative disorders? Neurosci. Lett. [Epub ahead of print]. 10.1016/j.neulet.2017.06.050 28668382

[B245] Waldemar G. Dubois B. Emre M. Georges J. McKeith I. G. Rossor M.. (2007). Recommendations for the diagnosis and management of Alzheimer's disease and other disorders associated with dementia: EFNS guideline. Eur. J. Neurol. 14, e1–e26. 10.1111/j.1468-1331.2006.01605.x 17222085

[B246] Walsh C. Barrow S. Voronina S. Chvanov M. Petersen O. H. Tepikin A. (2009). Modulation of calcium signalling by mitochondria. Biochim. Biophys. Acta 1787, 1374–1382. 10.1016/j.bbabio.2009.01.007 19344663

[B247] Waltenberger B. Mocan A. Smejkal K. Heiss E. H. Atanasov A. G. (2016). Natural Products to counteract the epidemic of cardiovascular and metabolic disorders. Molecules 21, 1–33. 10.3390/molecules21060807 27338339 PMC4928700

[B248] Wan W. Cao L. Liu L. Kalionis B. Chen C. Tai X.. (2014). EGb761 provides a protective effect against Aβ(1-42) oligomer-induced cell damage and blood-brain barrier disruption in an *in vitro* bEnd.3 endothelial model. PLoS ONE 9:e113126. 10.1371/journal.pone.0113126 25426944 PMC4245095

[B249] Wang C. Wang B. (2016). *Ginkgo biloba* extract attenuates oxidative stress and apoptosis in mouse cochlear neural stem cells. Phytother. Res. 30, 774–780. 10.1002/ptr.5572 26799058

[B250] Wang C. Youle R. J. (2009). The Role of Mitochondria in Apoptosis. Annu. Rev. Genet. 43, 95–118. 10.1146/annurev-genet-102108-134850 19659442 PMC4762029

[B251] Wang D.-S. Dickson D. W. Malter J. S. (2006). beta-Amyloid degradation and Alzheimer's disease. J. Biomed. Biotechnol. 2006:58406. 10.1155/JBB/2006/58406 17047308 PMC1559921

[B252] Wang P. Su C. Li R. Wang H. Ren Y. Sun H.. (2014). Mechanisms and effects of curcumin on spatial learning and memory improvement in APPswe/PS1dE9 mice. J. Neurosci. Res. 92, 218–231. 10.1002/jnr.23322 24273069

[B253] Wang Y.-H. Du G.-H. (2009). Ginsenoside Rg1 inhibits beta-secretase activity *in vitro* and protects against Abeta-induced cytotoxicity in PC12 cells. J. Asian Nat. Prod. Res. 11, 604–612. 10.1080/10286020902843152 20183297

[B254] Wang Y. Huang L. Tang X. Zhang H. (2010). Retrospect and prospect of active principles from Chinese herbs in the treatment of dementia. Acta Pharmacol. Sin. 31, 649–664. 10.1038/aps.2010.46 20523337 PMC4002969

[B255] Wang Y. Yang G. Gong J. Lu F. Diao Q. Sun J.. (2016). Ginseng for Alzheimer's disease: a systematic review and meta-analysis of randomized controlled trials. Curr. Top. Med. Chem. 16, 529–536. 10.2174/1568026615666150813143753 26268331

[B256] Weksler M. E. Szabo P. Relkin N. R. Reidenberg M. M. Weksler B. B. Coppus A. M. W. (2013). Alzheimer's disease and Down's syndrome: treating two paths to dementia. Autoimmun. Rev. 12, 670–673. 10.1016/j.autrev.2012.10.013 23201920

[B257] Winblad B. Amouyel P. Andrieu S. Ballard C. Brayne C. Brodaty H.. (2016). Defeating Alzheimer's disease and other dementias: a priority for European science and society. Lancet Neurol. 15, 455. 10.1016/S1474-4422(16)00062-4 26987701

[B258] Wolters B. (1994). Drogen, Pfeilgift und Indianermedizin: Arzneipflanzen aus Südamerika. Greifenberg: Urs Freud Verlag GmbH.

[B259] Xiong Z. Hongmei Z. Lu S. Yu L. (2011). Curcumin mediates presenilin-1 activity to reduce beta-amyloid production in a model of Alzheimer's Disease. Pharmacol. Rep. 63, 1101–1108. 10.1016/S1734-1140(11)70629-6 22180352

[B260] Yamada T. Terashima T. Honma H. Nagata S. Okubo T. Juneja L. R.. (2008). Effects of theanine, a unique amino acid in tea leaves, on memory in a rat behavioral test. Biosci. Biotechnol. Biochem. 72, 1356–1359. 10.1271/bbb.70669 18460792

[B261] Yan D. Zhang Y. Liu L. Yan H. (2016). Pesticide exposure and risk of Alzheimer's disease: a systematic review and meta-analysis. Sci. Rep. 6:32222. 10.1038/srep32222 27581992 PMC5007474

[B262] Yan F.-L. Zheng Y. Zhao F.-D. (2008). Effects of *Ginkgo biloba* extract EGb761 on expression of RAGE and LRP-1 in cerebral microvascular endothelial cells under chronic hypoxia and hypoglycemia. Acta Neuropathol. 116, 529–535. 10.1007/s00401-008-0435-6 18830615

[B263] Yan J. Hu J. Liu A. He L. Li X. Wei H. (2017). Design, synthesis, and evaluation of multitarget-directed ligands against Alzheimer's disease based on the fusion of donepezil and curcumin. Bioorg. Med. Chem. 25, 2946–2955. 10.1016/j.bmc.2017.02.048 28454848

[B264] Yanagisawa D. Taguchi H. Yamamoto A. Shirai N. Hirao K. Tooyama I. (2011). Curcuminoid binds to amyloid-beta1-42 oligomer and fibril. J. Alzheimers. Dis. 24(Suppl. 2), 33–42. 10.3233/JAD-2011-102100 21335654

[B265] Yang E.-J. Min J. S. Ku H.-Y. Choi H.-S. Park M. Kim M. K.. (2012). Isoliquiritigenin isolated from Glycyrrhiza uralensis protects neuronal cells against glutamate-induced mitochondrial dysfunction. Biochem. Biophys. Res. Commun. 421, 658–664. 10.1016/j.bbrc.2012.04.053 22538371

[B266] Yang L. Hao J. Zhang J. Xia W. Dong X. Hu X.. (2009). Ginsenoside Rg3 promotes beta-amyloid peptide degradation by enhancing gene expression of neprilysin. J. Pharm. Pharmacol. 61, 375–380. 10.1211/jpp.61.03.0013 19222911

[B267] Ye J. Zhang Y. (2012). Curcumin protects against intracellular amyloid toxicity in rat primary neurons. Int. J. Clin. Exp. Med. 5, 44–49. 22328947 PMC3272685

[B268] Ye R. Zhang X. Kong X. Han J. Yang Q. Zhang Y.. (2011). Ginsenoside Rd attenuates mitochondrial dysfunction and sequential apoptosis after transient focal ischemia. Neuroscience 178, 169–180. 10.1016/j.neuroscience.2011.01.007 21219973

[B269] Yim S. B. Park S. E. Lee C. S. (2007). Protective effect of glycyrrhizin on 1-methyl-4-phenylpyridinium-induced mitochondrial damage and cell death in differentiated PC12 cells. J. Pharmacol. Exp. Ther. 321, 816–822. 10.1124/jpet.107.119602 17314199

[B270] Yin S. Y. Kim H. J. Kim H.-J. (2013). A comparative study of the effects of whole red ginseng extract and polysaccharide and saponin fractions on influenza A (H1N1) virus infection. Biol. Pharm. Bull. 36, 1002–1007. 10.1248/bpb.b13-00123 23727921

[B271] Yokota T. Nishio H. Kubota Y. Mizoguchi M. (1998). The inhibitory effect of glabridin from licorice extracts on melanogenesis and inflammation. Pigment cell Res. 11, 355–361. 10.1111/j.1600-0749.1998.tb00494.x 9870547

[B272] Yun T. K. (2001). Brief introduction of Panax ginseng C.A. Meyer. J. Korean Med. Sci. 16(Suppl.) S3–S5. 10.3346/jkms.2001.16.S.S3 11748372 PMC3202213

[B273] Zhang L. Fang Y. Xu Y. Lian Y. Xie N. Wu T.. (2015). Curcumin improves amyloid beta-peptide (1-42) induced spatial memory deficits through BDNF-ERK signaling pathway. PLoS ONE 10:e0131525. 10.1371/journal.pone.0131525 26114940 PMC4482657

[B274] Zhang L. Fiala M. Cashman J. Sayre J. Espinosa A. Mahanian M.. (2006). Curcuminoids enhance amyloid-beta uptake by macrophages of Alzheimer's disease patients. J. Alzheimers. Dis. 10, 1–7. 10.3233/JAD-2006-10101 16988474

[B275] Zhou J.-S. Wang J.-F. He B.-R. Cui Y.-S. Fang X.-Y. Ni J.-L.. (2014). Ginsenoside Rd attenuates mitochondrial permeability transition and cytochrome c release in isolated spinal cord mitochondria: involvement of kinase-mediated pathways. Int. J. Mol. Sci. 15, 9859–9877. 10.3390/ijms15069859 24897022 PMC4100126

[B276] Zhou S. Lim L. Y. Chowbay B. (2004). Herbal modulation of P-glycoprotein. Drug Metab. Rev. 36, 57–104. 10.1081/DMR-120028427 15072439

[B277] Zhou X. Cui G. Tseng H. H. L. Lee S. M.-Y. Leung G. P. H. Chan S. W.. (2016a). Vascular contributions to cognitive impairment and treatments with traditional chinese medicine. Evid. Based. Complement. Alternat. Med. 2016, 9627258. 10.1155/2016/9627258 28042305 PMC5141557

[B278] Zhou X. Seto S. W. Chang D. Kiat H. Razmovski-Naumovski V. Chan K.. (2016b). Synergistic effects of chinese herbal medicine: a comprehensive review of methodology and current research. Front. Pharmacol. 7:201. 10.3389/fphar.2016.00201 27462269 PMC4940614

[B279] Zhou X. Wang H.-Y. Wu B. Cheng C.-Y. Xiao W. Wang Z.-Z.. (2017). Ginkgolide K attenuates neuronal injury after ischemic stroke by inhibiting mitochondrial fission and GSK-3beta-dependent increases in mitochondrial membrane permeability. Oncotarget 8, 44682–44693. 10.18632/oncotarget.17967 28591721 PMC5546510

[B280] Zhu X. Chen C. Ye D. Guan D. Ye L. Jin J.. (2012). Diammonium glycyrrhizinate upregulates PGC-1α and protects against Aβ 1–42-induced neurotoxicity. PLoS ONE 7:e35823. 10.1371/journal.pone.0035823 22540007 PMC3335163

[B281] Zhu X. Perry G. Smith M. A. Wang X. (2013). Abnormal mitochondrial dynamics in the pathogenesis of Alzheimer's disease. J. Alzheimers. Dis. 33(Suppl. 1), S253–S262. 10.3233/JAD-2012-129005 22531428 PMC4097015

[B282] Zlokovic B. V. (2011). Neurovascular pathways to neurodegeneration in Alzheimer's disease and other disorders. Nat. Rev. Neurosci. 12, 723–738. 10.1038/nrn3114 22048062 PMC4036520

[B283] Zupancic M. Mahajan A. Handa K. (2011). Dementia with lewy bodies: diagnosis and management for primary care providers. Prim. Care Companion CNS Disord. 13:PCC.11r01190. 22295275 10.4088/PCC.11r01190 PMC3267516

